# Supplementary material for: COVID-19 and mental health in 8 low- and middle-income countries: A prospective cohort study
Source: PLoS Med. 2023 Apr 6;20(4):e1004081. doi: 10.1371/journal.pmed.1004081 (PMC10079130; doi:10.1371/journal.pmed.1004081)
Supplement: S1 Fig — Figure plots the data collection and lean season timeline across samples to show which crop cycle the COVID-19 arrived in the countries. The y-axis shows the sample. The x-axis shows the calendar time. Dark blue color refers to individual surveys during the data collection, while dark orange color shows the main lean season across countries and years. The red vertical line shows the onset of the COVID-19 pandemic on 11 March 2020. Data are retrieved from the FAO Global Information and Early Warning System. For further information about definitions, please see the FAO’s website. (PDF) [file pmed.1004081.s001.pdf]

S1 Fig. Data collection and lean season timeline

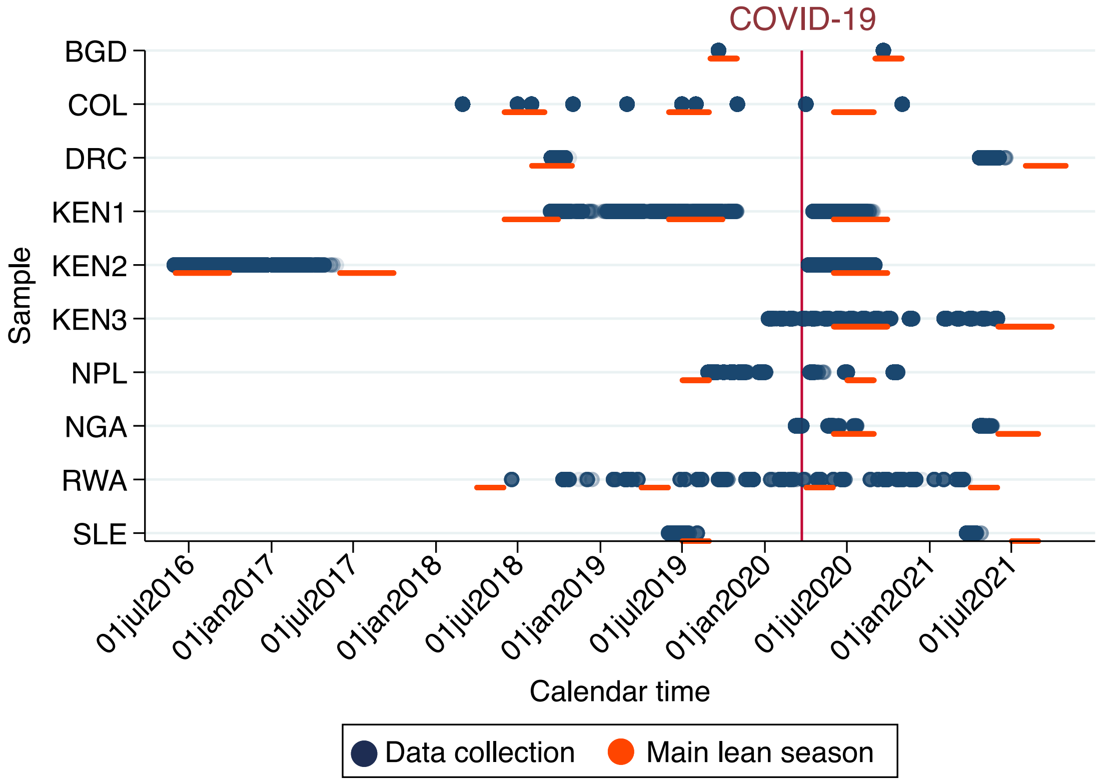

Notes. Figure plots the data collection and lean season timeline across samples to show which crop cycle the COVID-19 arrived in the countries. The Y-axis shows the sample. The X-axis shows the calendar time. Dark blue color refers to individual surveys during the data collection while dark orange color shows the main lean season across countries and years. The red vertical line shows the onset of the COVID-19 pandemic on 11 March 2020.The red vertical line shows the onset of the COVID-19 pandemic on 11 March 2020. Data are retrieved from the FAO Global Information and Early Warning System (2022). For further information about definitions please see the FAO's website.

References

1. The Food and Agriculture Organization. Global Information and Early Warning System. 2022. Available from: `https://www.fao.org/giews/countrybrief/index.jsp`.
