# Supplementary material for: COVID-19 and mental health in 8 low- and middle-income countries: A prospective cohort study
Source: PLoS Med. 2023 Apr 6;20(4):e1004081. doi: 10.1371/journal.pmed.1004081 (PMC10079130; doi:10.1371/journal.pmed.1004081)
Supplement: S2 Fig — (PDF) [file pmed.1004081.s002.pdf]

**S2 Fig. Mental Health, Lockdowns, and Cases**

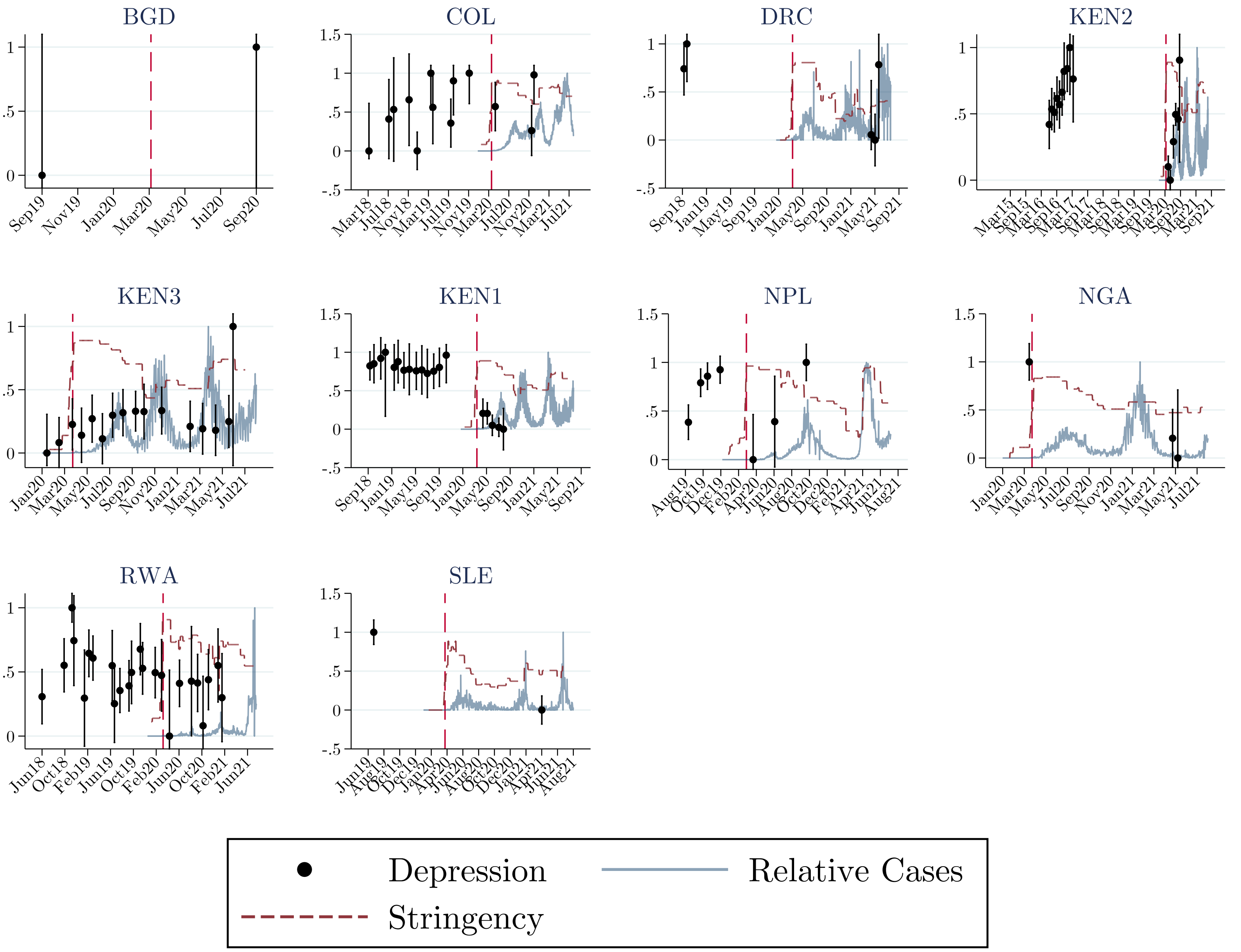


Figure shows relative levels of unweighted depression index, national COVID-19 cases per-capita, and national policy stringency index, for each of our samples over time. All three series are standardized so that all values fall between 0 and 1. The black points and error bars show predicted values and 95% confidence intervals for the depression index. The blue solid line shows new daily COVID-19 cases per-capita. The red dashed line shows values of the COVID-19 policy stringency index.
