# Supplementary material for: COVID-19 and mental health in 8 low- and middle-income countries: A prospective cohort study
Source: PLoS Med. 2023 Apr 6;20(4):e1004081. doi: 10.1371/journal.pmed.1004081 (PMC10079130; doi:10.1371/journal.pmed.1004081)
Supplement: S3 Fig — Figure plots the average change in mental health pre-post COVID across samples using a common estimation strategy that uses individual fixed-effects without additional controls for all samples. The y-axis is the change in mental health relative to pre-COVID. The x-axis shows time after the onset of COVID discretized into 2 groups: 0 to 4 months and more than 4 months. Point estimates are represented as dots with 95% confidence intervals as whiskers. The color of the points indicates which method of weighting our indices was used: the factor loadings (black, left), the unweighted index (dark gray, middle), or the inverse-covariance weights (light gray, right). The average effect sizes and confidence intervals are estimated from a random-effects model using restricted maximum likelihood to estimate the variance in true effects across samples. (PDF) [file pmed.1004081.s003.pdf]

S3 Fig. Random Effects Aggregates With Individual Fixed Effects and No Additional Controls

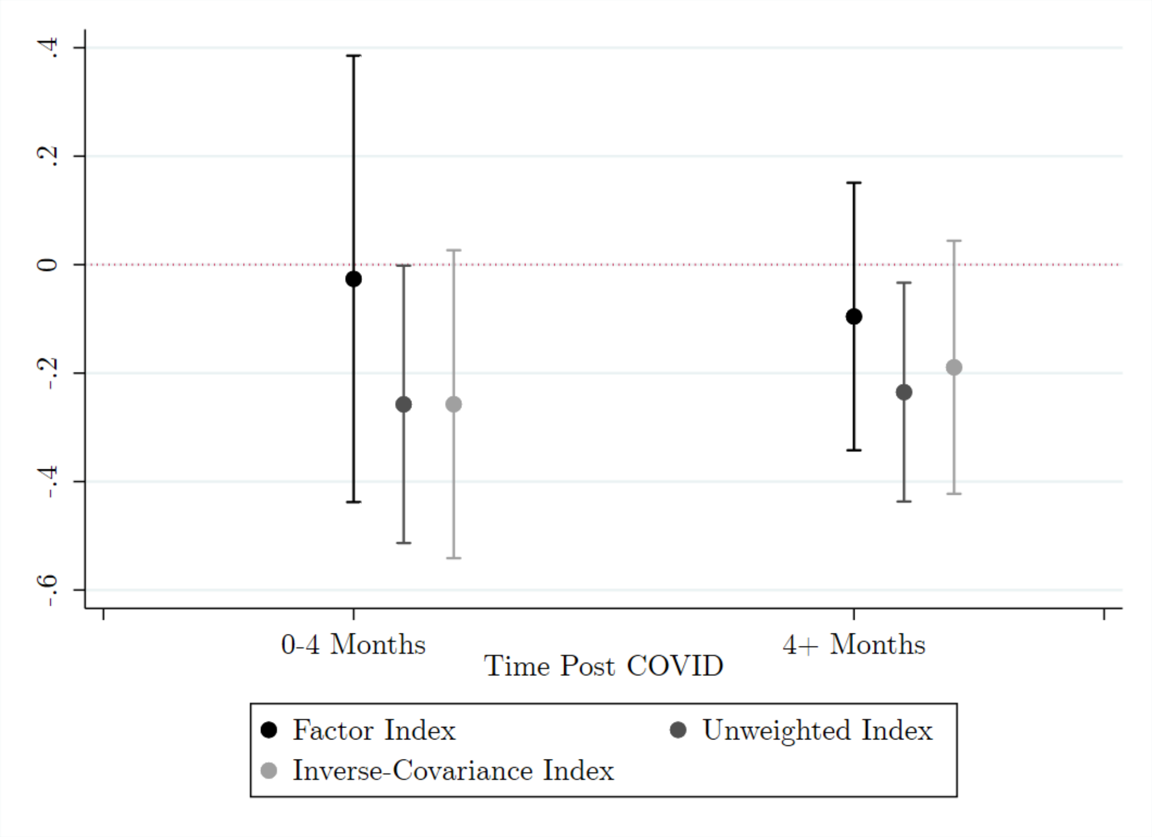

Notes. Figure plots the average change in mental health pre-post COVID across samples using a common estimation strategy that uses individual fixed-effects without additional controls for all samples. The Y-axis is the change in mental health relative to pre-COVID. The X-axis shows time after the onset of COVID discretized into two groups: 0-4 months, and more than 4 months. Point estimates are represented as dots with 95% confidence intervals as whiskers. The color of the points indicates which method of weighting our indices was used: the factor loadings (black, left), the unweighted index (dark gray, middle) or the inverse-covariance weights (light gray, right). The average effect sizes and confidence intervals are estimated from a random-effects model using restricted maximum likelihood to estimate the variance in true effects across samples.
