# Supplementary material for: COVID-19 and mental health in 8 low- and middle-income countries: A prospective cohort study
Source: PLoS Med. 2023 Apr 6;20(4):e1004081. doi: 10.1371/journal.pmed.1004081 (PMC10079130; doi:10.1371/journal.pmed.1004081)
Supplement: S4 Fig — Figure shows estimates of the relationship between unweighted depression index and COVID-19 policies and cases. (PDF) [file pmed.1004081.s004.pdf]

(a) Independent Variables Aggregated by Week

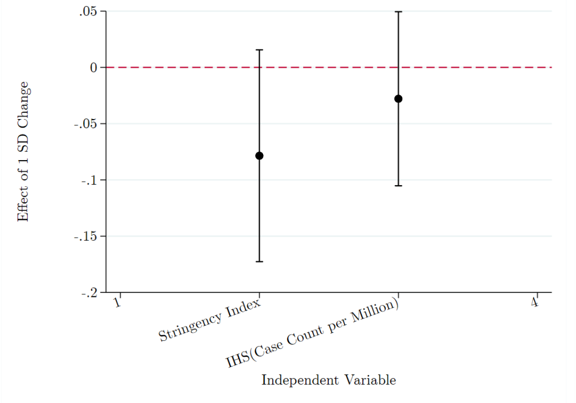

(b) Independent Variables Aggregated by Fortnight

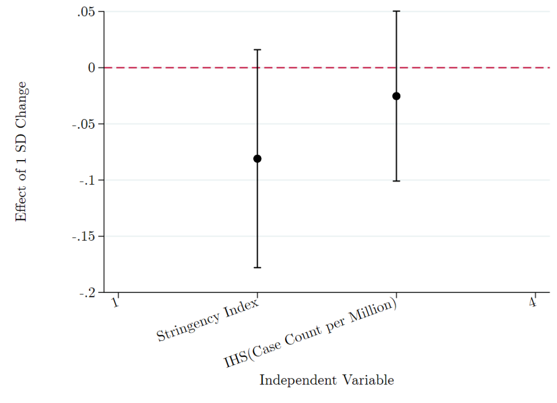

(c) Independent Variables Aggregated by Month

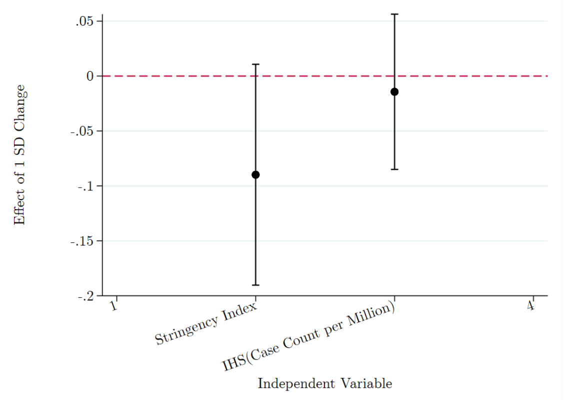

S4 Fig. Relationship between COVID-19 Policies, Cases, and Depression

Note: Figure shows estimates of the relationship between unweighted depression index and COVID-19 policies and cases. Methodology for these estimates can be found in **S5 Appendix**.
