# Supplementary material for: COVID-19 and mental health in 8 low- and middle-income countries: A prospective cohort study
Source: PLoS Med. 2023 Apr 6;20(4):e1004081. doi: 10.1371/journal.pmed.1004081 (PMC10079130; doi:10.1371/journal.pmed.1004081)
Supplement: S5 Fig — Figure shows regression discontinuity estimates of change in depression following onset of the pandemic with varying bandwidths used in estimation. The x-axis shows the share of the available pre-COVID time period used in each sample used in estimation. In each estimate, the sample was reduced so that only the final D days of data prior to and after the onset of the pandemic were used, where D was set to be a certain percentage of the range of the data prior to the onset of the pandemic. We aggregate the results for each percentage of the available time used using the same random-effects model used in Fig 2. (PDF) [file pmed.1004081.s005.pdf]

(a) Aggregate Estimate

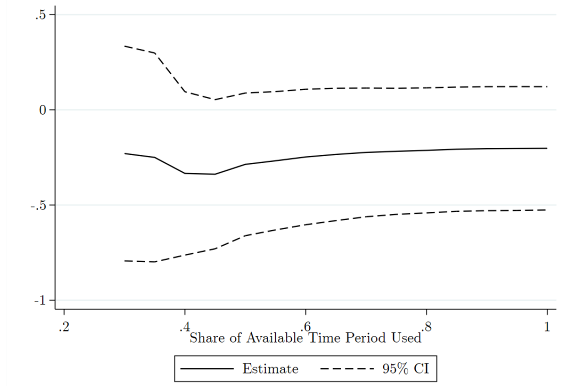

(b) Sample-Specific Estimates

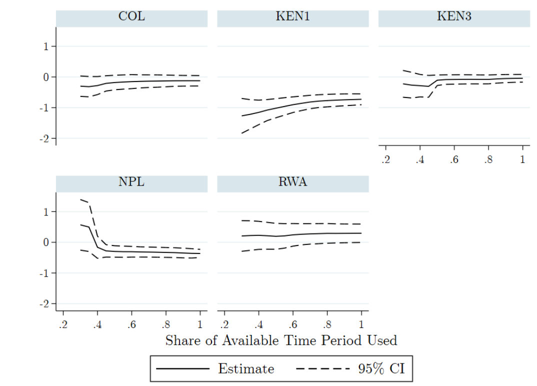

S5 Fig. Sensitivity of RD Estimates to Time Duration Used in Estimation

Note: Figure shows regression discontinuity estimates of change in depression following onset of the pandemic with varying bandwidths used in estimation. The x-axis shows the share of the available pre-COVID time period used in each sample used in estimation. In each estimate, the sample was reduced so that only the final **D** days of data prior to and after the onset of the pandemic were used, where **D** was set to be a certain percentage of the range of the data prior to the onset of the pandemic. We aggregate the results for each percentage of the available time used using a random-effects model.
