# Supplementary material for: COVID-19 and mental health in 8 low- and middle-income countries: A prospective cohort study
Source: PLoS Med. 2023 Apr 6;20(4):e1004081. doi: 10.1371/journal.pmed.1004081 (PMC10079130; doi:10.1371/journal.pmed.1004081)
Supplement: S6 Fig — Figure shows a graphical representation of the regression discontinuity (RD) estimates for each of the 5 samples in Table 2. The x-axis is the running variable in the RD design, days post onset of lockdowns in that country. The y-axis is standard deviation units of our unweighted depression index. The points are the average values of the index within discrete time bins. The solid lines are linear fits to the points shown pre and post the onset of the lockdown. (PDF) [file pmed.1004081.s006.pdf]

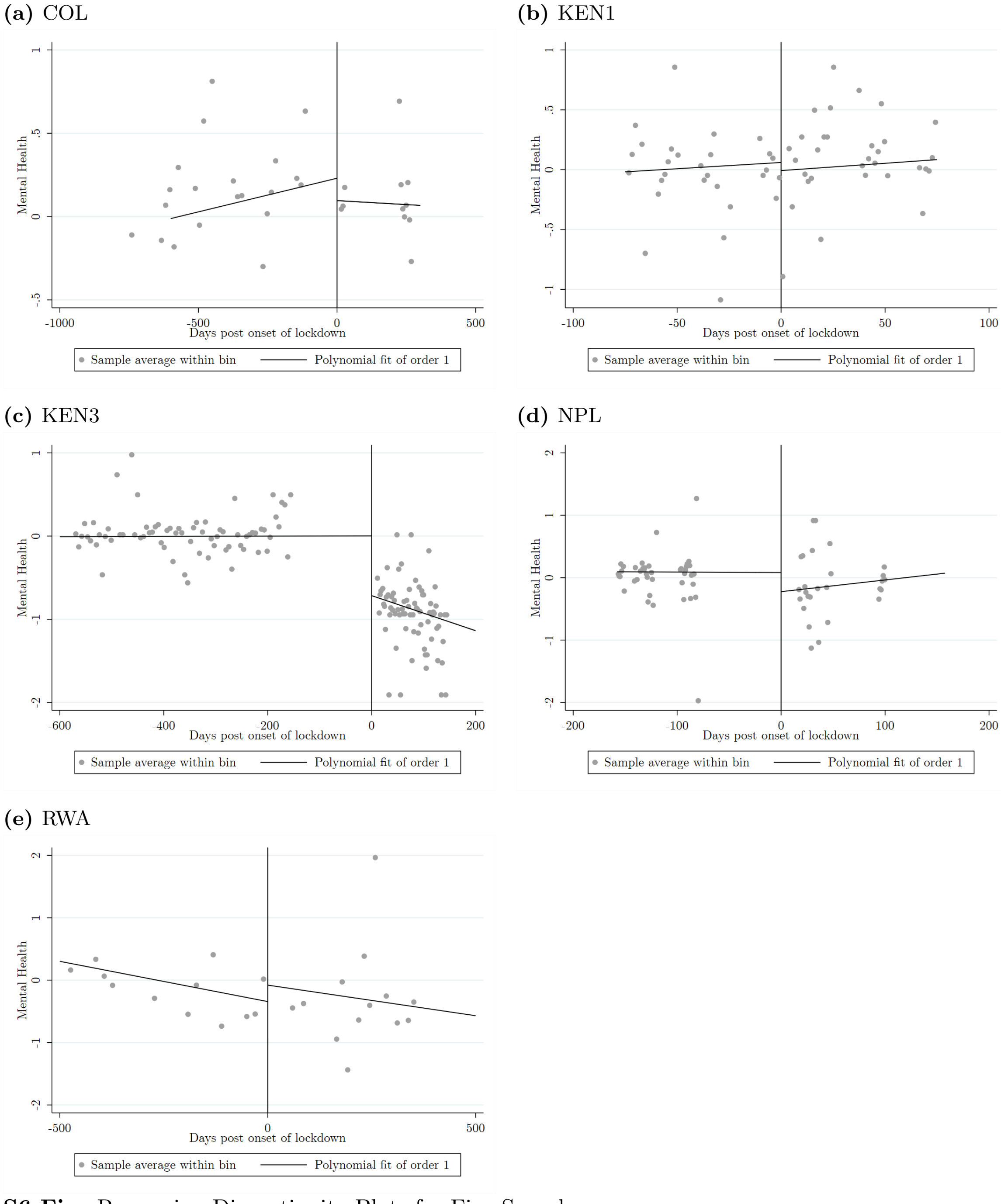

S6 Fig. Regression Discontinuity Plots for Five Samples

Figure shows a graphical representation of the regression discontinuity (RD) estimates for each of the five samples in **Table 2**. The x-axis is the running variable in the RD design, days post onset of lockdowns in that country. The y-axis is standard deviation units of our unweighted depression index. The points are the average values of the index within discrete time bins. The solid lines are linear fits to the points shown pre and post the onset of the lockdown.
