# Supplementary material for: COVID-19 and mental health in 8 low- and middle-income countries: A prospective cohort study
Source: PLoS Med. 2023 Apr 6;20(4):e1004081. doi: 10.1371/journal.pmed.1004081 (PMC10079130; doi:10.1371/journal.pmed.1004081)
Supplement: S1 Appendix — (PDF) [file pmed.1004081.s007.pdf]

S1 Appendix. Sample description

This Appendix section describe the economic context, COVID-19-related developments, and sample construction for each sample in the study.

Bangladesh

COVID-19 Experience

Case History:

- March 8, 2020: First confirmed cases reported by the Institute of Epidemiology, Disease Control, and Research (IEDCR)
- Total cases: 1,071,774 as of July 15, 2021 [1]
- Total deaths: 17,278 as of July 15, 2021 [1]

Mobility Restrictions:

- March 14, 2020: On-arrival visas suspended for all countries. Ban on flights from all European countries except the United Kingdom
- March 26-May 30, 2020: The Government of Bangladesh (GoB) declared a "national holiday", limited the availability of public transport, and ordered all public and private offices to remain closed. Only food markets, pharmacies, hospitals, and emergency services were allowed to remain open.
- April 9, 2020: The GoB imposes a "complete lockdown" on Cox's Bazar District. No entry and exit from the district is permitted.
- June 1, 2020: The GoB divides the country into zones (high risk, moderate, and low risk), based on the number of COVID-19 cases. Movement across areas was restricted.

Social Distancing:

- May 31, 2020: Face masks mandatory when outside the home.
- June 27, 2020: Public gatherings prohibited in red zones. Stay at home required for red zones.

School Closures:

- March 17, 2020-September 13, 2021: Closure of all schools and universities.

Social Protection Responses: Bangladesh launched two major cash transfer programs in response to the pandemic. The assistance program for garment sector workers offered digital payments to 4 million employees at textile factories [2], [3]. Another cash transfer program offered top-up payments to 5 million households who were already receiving government benefits [4]. These two programs benefitted approximately 15% of the population [2].

- March 26: Prime Minister announced an USD 588 million package for export-oriented industries, to be spent on employee salaries

- April 5: Prime Minister announced an USD 8 billion stimulus package for hard-hit industries, small and medium enterprises (SMEs), and emergency incentives for export oriented industries. The GoB announced an expansion of the social safety net programs—including the Vulnerable Group Feeding(VGF) and Vulnerable Group Development (VGD) programs—and reductions in rice prices.

Economic Context

Seasonality and Food security The Bangladesh survey was conducted in September 2019 and October 2020, which partially overlaps with the lean period. Despite an above-average 2019 harvest, prices of rice during in Bangladesh survey remained well above their prior year levels, linked to pandemic-related increases in demand and concerns about the upcoming 2020 harvest [18].

The Bangladesh post-COVID surveys in this article were conducted after to Tropical Cyclone Amphan, which struck southwestern parts of Bangladesh on 20 May 2020 and caused loss of life and substantial devastation, including livestock and crops.

Refugees The over 900,000 Rohingya refugees living primarily in Cox's Bazar district since 2017 have experienced food insecurity and required humanitarian assistance to meet daily needs since well prior to the Covid-19 pandemic [18].

Social Protection The social protection policy environment in Bangladesh is fragmented, with over 114 separate programs providing cash and food transfers to the vulnerable [5].

Bangladesh BGD, Rohingya refugees from Myanmar: Refugee camp households in Bangladesh

Project Title: Mental wellbeing of displaced Rohingya women: a cluster randomised controlled trial (RCT)

Target Population: Rohingya women with a child aged 0-2 years.

Original Study Design: Cluster RCT.

Sampling Frame: Geographic-level information, which is blocks within the camps, was considered for randomisation. At the time of randomization, there were about 2000 blocks. The number of blocks required in each arm was computed with an improvement in mental health by 0.05 units (on a mental health index scale between 0 and 1) at 5% significance level with 80% power. A minimum of 112 blocks with 1,568 mother-child pairs are required in each arm to meet this requirement. A total of 251 blocks were selected, of which 137 were assigned to the treatment (54%) and 114 were assigned to the control group (46%). Treatment and control blocks have been selected in a manner that they were not too close to each other, minimizing potential contamination.

COVID-19 Survey Design: Phone surveys.

Sampling Frame: At baseline, a total of 3499 participants were surveyed: 1,911 from the treatment and 1,588 from the control groups. At endline, 2,845 participants were surveyed, 1,679 from the treatment and 1,166 from the control arm.

Survey Dates: Baseline in September 2019; endline in October 2020.

Sample size, tracking and attrition: About 19% participants attired, mostly due to lack of access to mobile phones.

Median survey time: Median survey time is unknown as survey was conducted over the phone and there were other components on child health.

Sampling Weights: N/A.

IRB Approval: This research was approved by BRAC University (ref no. 2019-028-ER).

Colombia

COVID-19 Experience

Case History:

- March 6, 2020: First confirmed case
- Total cases: 4,583,442 as of July 15, 2021 [1]
- Total deaths: 114,833 as of July 15, 2021 [1]

Mobility Restrictions:

- March 17-July 1, 2020: Borders closed.
- March 24, 2020: Mandatory preventive isolation implemented throughout the country, which allows one member of the family to leave to buy food, medicine and carry out financial transactions (extended to September 1).
- March 24, 2020: General lockdown until April 13 (extended to September 1).
- May 6, 2020: Three exceptions for internal movement: i) humanitarian emergency; ii) transport of cargo or merchandise; iii) fortuitous event or force majeure.

School Closures:

- March 24, 2020: Schools are closed.

Social Distancing:

- March 16, 2020: Public events for more than 50 people are banned.
- March 24, 2020: Mandatory preventive isolation implemented throughout the country. Social events and activities are prohibited including religious services involving crowds or gatherings, group sports, gyms, bars and discos, and cinemas and theaters. Restaurants can only provide take-away orders. Consumption of alcohol in open spaces is banned.
- April 4, 2020: The Government mandates the use of face masks in public transit and in areas of high volume such as supermarkets, banks and pharmacies. Face masks are mandatory for people with respiratory symptoms and vulnerable groups such as adults over 70.

Social Protection Responses:

- March 25, 2020: Individuals in strata 1 and 2 can defer payment of energy and gas bills up to 36 months during mandatory preventive isolation. Cash transfers and food aid provided to households, youth and the elderly.

- In March 2020, Colombia rolled out a new unconditional cash transfer program, *Devolución del IVA*, benefitting one million low-income households. The transfer is paid every two months to recipients of *Familias en Acción*, and *Colombia Mayor*. The 75,000 peso (USD 20) additional transfer represents 8% of the monthly minimum wage. Approximately 27% of the population has received this cash transfer program [2].

Economic Context

Seasonality and Food Security The Colombia post-COVID survey was conducted during the beginning of a lean period. The price of rice reached a record high in April 2020, shortly after COVID-19 hit the region. Prices remained 40% above year-earlier values by June 2020.

Conflict The Coordination Platform for Refugees and Migrants for Venezuela estimated that nearly 5 million people have fled from the country as of mid-March 2020. Colombia is the main host country of refugees and migrants from Venezuela (Bolivarian Republic of Venezuela), with an estimated population of nearly 1.8 million. According to a February 2020 World Food Program survey, more than 20 percent of the migrant population in the four departments that border with Venezuela were severely food insecure. Migrant households have minimal access to employment, increasing food insecurity. On top of the inflow of Venezuelan refugees, Colombia has 8.2 million Internally Displaced Persons, which accounts to 16% of the population and is the highest number of IDP worldwide. Despite a generous and progressive framework that started in 1997, IDP are still more vulnerable than the urban and rural poor. In 2014, poverty and extreme poverty rates were 1.7 and 3.5 times higher for IDP relative to national averages. Conflict and force displacement are still happening and during the pandemic (between 2020 and 2021), internal forced displacement rose by 181%.

Social Protection Colombia provides several social protection programs, including a

conditional cash transfer program, *Familias en Acción*, and *Colombia Mayor*, a non-contributory pension program for low-income senior citizens.

Colombia COL, Primary caregivers in Tumaco, Colombia

Project Title: Semillas de Apego: a psychosocial group model to promote maternal mental health and early childhood development

Target Population: Primary caregivers of young children (ages 3-5) enrolled in public Early Childhood Development Centers in Tumaco, Colombia. Caregivers had a prior exposure to violence and forced displacement.

Original Study Design: Original study is based on cluster-based randomization to the 'Semillas de Apego' psychosocial support program. ECDCs were first randomised to the treatment or control groups. Second, all caregivers of children who were attending an ECDC were randomly allocated to four sequential cohorts following a phased-in approach. All caregivers received regular child and family services provided by the ECDC, while caregivers in the treatment group received an invitation to participate in the program. In every cohort, participants completed assessments at baseline, and 1 and 8-month follow-ups after the program had been completed with the treatment group. Baseline survey included a detailed socioeconomic module, questionnaire on exposure to violence and forced displacement, and scales on child-parent interactions and early-childhood development. Follow-up surveys included the latter scales and short modules to record changes in socioeconomic characteristics and exposure to violence between waves.

Sampling Frame: Universe of caregivers whose children were served by 18 public ECDC in Tumaco, Nariño. These 18 ECDC serve 80% of the 1600 families in the municipality whose children are enrolled across public ECDCs. Sampling in each cohort was designed using administrative-level data provided by each ECDC. 1376 primary caregivers participated over the course of the programme in the treatment (n=714) or control (n=662) groups.

COVID-19 Survey Design: Phone surveys with selected items from SCL-90-R battery administered in in-person data collection, Parenting Stress Index short form questionnaire, and a module on Covid-related stressors.

Sampling Frame: Sample of 1376 caregivers who had participated in the prior

(pre-pandemic) RCT and data collection.

Survey Dates: Baseline data was collected in March, 2018 (Cohort 1); July, 2018 (Cohort 2); March, 2019 (Cohort 3); and July, 2019 (Cohort 4). 1-month follow-up data was collected in August, 2018 (Cohort 1); November, 2018 (Cohort 2); August, 2019 (Cohort 3); and November, 2019 (Cohort 4). 8-month follow-up data was collected in Marc, 2019 (Cohort 1); and August, 2018 (Cohort 2) and was scheduled to be collected in March, 2020 (Cohort 3); and August, 2020 (Cohort 4) but field work was interrupted because of the pandemic and associated lockdowns. Two short phone surveys were then administered to assess the mental health impact of the pandemic. First phone survey was administered between April 7 and 21, 2020 (Cohorts 3 and 4) and the second one between November 12 - December 12, 2020 (All cohorts).

Sample size, tracking and attrition: 1,376 caregivers. 90% (1245 of 1376) participants completed the 8-month follow-up survey (in-person or April phone survey). Attrition was not different between the in person (9%) and phone survey (10%) and was not predicted by baseline characteristics. 7% (1285 of 1376) participants completed the November 2020 phone survey.

Median survey time: In person surveys (including child-development battery): 2.5 hours. Phone surveys: 20 minutes.

Sampling Weights: N/A.

IRB Approval: Original study design approved by the Ethics Committee of the Universidad de los Andes, Colombia (protocol 786, 2017). Inclusion of Covid-19 surveys approved under the same study protocol in April 2020.

Democratic Republic of Congo

COVID-19 Experience

Case History:

- March 14, 2020: First confirmed case
- Total cases: 45,211 as of July 15, 2021 [1]
- Total deaths: 984 as of July 15, 2021 [1]

Mobility Restrictions:

- March 21, 2020: All borders are closed and international passenger flights are suspended [9].
- March 28, 2020: Places of worship, bars and nightclubs are closed.
- March 28, 2020: Workplaces closing except for workers providing essential goods and services.
- March 30, 2020: Lockdown measures include suspension of non-essential travel and a curfew until May 31, 2020 [6].
- May 18, 2020: Nationwide overnight curfew between 8pm and 5am, revised to 10pm to 5am in July 28, 2020, ended in August 15, 2020.

School Closures:

- March 28, 2020: Schools and higher education institutions are closed.
- May 18, 2020: Schools are reopened for primary schools' final year class and graduation class [7].
- June 1, 2020: The school grades of CM2, 3ieme and Terminale are reopened.
- August 10, 2020: All schools are reopened [8].

Social Distancing:

- March 28, 2020: Gatherings of more than 50 people are prohibited.
- August 15, 2020: Places of worship were allowed to reopen [9].

Social Protection Responses:

- March 31, 2020: Free water and electricity for all households during the confinement period. Financial aid of 4 billion granted to households and people experiencing poverty. Government is replacing less than 50% of lost salary.

Economic Context

Seasonality and Food Security The DRC post-COVID surveys were conducted between April through June 2021, during the lean season. Crop output in 2020 was lower than typical owing to floods, pests, violence, and COVID-19 preventative efforts

[10]. Between April and July 2020, the prices of imported food goods rose dramatically. Decrease in cash crop exports as a consequence of poor demand from importing countries and a slowdown in trade flows as a result of COVID-19 resulted in a fall in foreign exchange revenues and prices to rise.

Conflict Weak governance and the prevalence of many armed groups have subjected Congolese civilians to widespread rape and sexual violence, massive human rights violations, and extreme poverty. Millions of civilians have been forced to flee the fighting: the United Nations estimates there are currently 4.5 million internally displaced persons in the DRC [11].

Social Protection In 2017, a strategic framework was established by the government to adopt a comprehensive national social protection policy. However, the programs remained fragmented, poorly funded, and failed to adequately meet the needs of the poorest and most vulnerable in a context of deep fragility. As of 2018, the poverty rate of the country was 63.4%.

Democratic Republic of Congo (DRC), Impact evaluation of Community Based Trauma Healing

Project Title: Impact evaluation of Community Based Trauma Healing in the Democratic Republic of Congo

Target Population: Households in 160 communities in Nyangezi, Katana and Walikale Health Zones in Eastern DRC

Original Study Design: To evaluate the impact of a Community Based Trauma Healing (CBTH) implemented in Eastern DRC, as part of a wider program that aims to reduce Gender Based Violence. CBTH aims to address communal trauma through community engagement, building social cohesion and exploring and deconstructing negative stereotypes. The program recruited local Community Trauma Healers that were trained and supported by an international NGO to organize a community sessions and celebrations. During the session, the program aimed to help community members heal from post-conflict trauma resulting from, among other things, death, injury, (systematic) rape, indigenous conflict, internal displacement and inheritance conflicts. These community "detraumatisme" sessions brought together (potential) victims, perpetrators, traditional leaders and faith-based leaders. CBTH teaches

community members strategies to control emotions, and reduce risks of GBV. We use a cluster randomized trial to evaluate the impact of CBTH in 80 randomly selected communities in Eastern DRC.

Sampling Frame: To select the villages, first, the implementing partner selected three health zones, and then created a list of 40 health areas that met three criteria: they were receiving wider program interventions, they were relatively accessible, and had not anticipated major security risks that might undermine or interrupt program and evaluation. Within this subset of health areas, 160 eligible villages were identified on the basis of their accessibility, relative security, population size and household count, and a limited presence of other projects that might contaminate any impact of the intervention. In each health area, the implementing partner identified four villages. Pre-COVID (baseline) data was collected during September and October 2018 each of the 160 villages. Within each village, field teams used a ”random-walk” methodology to randomly select households. Enumerators interviewed every 3rd household on the right in rural areas and every 5th household on the right in semi-urban areas, starting from a predetermined village landmark (market, administrative building, well, tree, etc.) Prior to the start of daily data collection, the team supervisor was in charge of dispersing enumerators around the village so that they could cover all the area and that the sample would not be confined to a small portion of the observation area. In each selected household, enumerators interviewed a random adult respondent (age 18 or over) selected from within the household (using a kish-grid method programmed on the tablet). As per the programming, and to ensure the highest level of responsiveness on sensitive topics, female enumerators only interviewed female respondents and male enumerators only interviewed male respondents. In total, we collected baseline data from 3678 respondents.

COVID-19 Survey Design: Post COVID (endline) data was collected during April through June 2021. We aimed to resurvey all baseline respondents. When enumerators were unable to interview a baseline respondent, they recorded the reason (unable to locate respondent, respondent temporarily away, permanently away, died, or refusal). If the enumerator was able to locate the household but not the specific baseline respondent in that household, then the enumerator attempted to find another eligible respondent (18 or older) of the same gender as the baseline respondent within

the household that was closest in age. Failing that, the enumerator would replace the household with the household to the right of the original household and would identify.

Sampling Frame: This study uses data from the control group only, comprising 80 villages, comprising 1414 respondents.

Survey Dates: September and October 2018 and April through June 2021.

Sample Size, Tracking and Attrition: Pre-COVID data was collected under 1771 respondents. Followup data was collected under 1771 respondents.

Median Survey Time: 67 minutes

Sampling Weights: None.

IRB Approval: The research protocol for this study was approved by the NORC Institutional Review Board (IRB00000967), Project Number 7554.059.01, IRB Protocol Number 18.08.13 on 09/10/2018.

Kenya

COVID-19 Experience

Case History:

- First confirmed case: March 13, 2020
- Total cases: 191,020 as of July 15, 2021 [1]
- Total deaths: 3,746 as of July 15, 2021 [1]

Mobility Restrictions:

- March 15, 2020: Entry restricted to citizens and foreigners with valid residence permits, who must self-quarantine upon arrival. Non-essential government and businesses directed to begin working from home.
- March 25, 2020: National and international flights halted.
- March 27, 2020: Start of dusk to dawn curfew (7pm-5am). Schedule changes to 9pm-4am on June 7; to 11pm–4am on September 28, and to 10pm-4am on November 4. Extended to October 20, 2021.
- April 6, 2020: Partial mobility restrictions begin; movement in and out of Nairobi Metropolitan Area restricted. On April 8, 2020, Mombasa, Kilifi and

Kwale were was added to this list. Mandera was added on April 22, 2020. Restrictions were lifted for Kilifi and Kwale on June 6, 2020; they remain in place for Nairobi, Mombasa and Mandera.

- In addition, from May 6, 2020 - June 6, 2020, movement into and out of the Eastleigh in Nairobi and Old Town in Mombasa was restricted.

Social Distancing:

- March 15, 2020: Limits on large gatherings, church attendance, closure of courts; Safaricom suspends mobile money transaction fees to encourage cashless transactions.
- April 12, 2020: Mask wearing in public spaces mandatory.
- July 6, 2020: Re-opening for congregational worship and public (in-person) worship.

School Closures:

- March 20, 2020 - January 4, 2021: Closure of all schools and higher education institutions.

Social Protection Responses:

- March 25, 2020: Announcement of tax breaks for households and enterprises, accelerated payments of bills and tax credits, additional appropriations for cash transfers to the elderly, orphans and vulnerable households by the Ministry of Labour and Social Protection.
- May 23, 2020: Stimulus package announced, including spending on infrastructure, tourism, education sector and SMEs.
- There are reports that beneficiaries of the old age pension (Inua Jamii) program have received additional payments during the pandemic [2], but other sources suggest that they only received their standard payments [12].
- A public works program temporarily employed 26,000 young people to clean and sanitize poor urban neighborhoods [13].

Economic Context

Seasonality and Food Security The three Kenya post-COVID surveys were conducted as the country entered the lean season, following a below average cereal crop harvest in 2019. A severe desert locust outbreak, the worst in 70 years, has affected large areas in the north and central pastoral areas of the country (KEN3), but households in two Kenya samples (KEN1 and KEN2) are mostly unaffected [18].

Social Protection Kenya has four major cash transfer programs for people who are disabled, elderly, vulnerable children, or at risk of seasonal hunger, coordinated under the National Safety Net Program [14]. Approximately 5.7% of the population benefits from these programs [15].

Kenya KEN1, Participants in child health and human capital interventions in Kenya

Project Title: Kenya Life Panel Survey (KLPS)

Target Population: KLPS is a longitudinal dataset of over 6,500 Kenyans that participated in randomized child health and human capital interventions and have since been followed into adulthood. Respondents attended primary school in Busia, Kenya; migrants continue to be tracked and the study sample now resides throughout Kenya, Uganda, and elsewhere. Data used here come from the fourth round (KLPS-4), over 20 years after the first intervention, and phone surveys conducted during the COVID-19 pandemic.

Original Study Design: The KLPS sample comprises of individuals who participated in one of two previous randomized NGO programs: the Primary School Deworming Program (PSDP), which provided deworming medication to primary school students during 1998–2003 (Miguel and Kremer 2004), and the Girls' Scholarship Program (GSP), which provided merit scholarships to upper primary school girls in 2001 and 2002 (Kremer, Miguel and Thornton 2009). Approximately 20% subset of these individuals also participated in the vocational training and cash grants programs (known as the Start-up Capital for Youth and Vocational Education Program, or SCY/Voced) during 2009-2014. Data used in this paper come from KLPS Round 4 (KLPS-4), which collected information roughly 20 years after the initial deworming treatment.

Sampling Frame: The KLPS sample is split into 2 representative waves for data collection; analyses here focus on "Wave 1" respondents, as these individuals were surveyed in-person from October 2018-February 2020 and again as part of COVID-19 phone surveys.

COVID-19 Survey Design: All KLPS respondents were targeted for phone surveys. The KLPS sample was split into five representative "batches", and each batch was surveyed in sequence in order to provide representative data over time. Phone surveys were shortened from in-person surveys, capturing information on household earnings, consumption aggregates, physical and mental health.

Sampling Frame: A representative sample of approximately 7,500 PSDP participants (out of 32,000 pupils) were selected for inclusion in KLPS, along with the fourth round of the KLPS (KLPS-4) focuses on the subsets of the KLPS sample who participated in the PSDP or SCY/Voced interventions.

Survey Dates: Previous KLPS survey rounds were conducted in 2003-05 (KLPS-1), 2007-09 (KLPS-2), and 2011-14 (KLPS-3). KLPS-4 E+ Module (economic data) were collected from 2017-19, and KLPS-4 Wave 1 I-Module surveys (health and mental health data) were conducted from October 2018 to February 2020. COVID-19 phone surveys were conducted from April to August 2020.

Sample size, tracking and attrition: KLPS-4 I Module Wave 1 sample size: 4,076. KLPS-4 I Module Wave 1 effective survey rate among the non-deceased (accounting for intensive tracking): 89.6%. COVID-19 phone survey rate among Wave 1 respondents: 77.5%.

Median survey time: In-person surveys: 146 min; Phone surveys: 37 min.

Sampling Weights: Observations are weighted to be representative of the original KLPS population.

IRB Approval: UC Berkeley and Maseno University.

Kenya KEN2, Rural Households in NGO Cash Transfer Study

Project Title: General Equillibrium Effects of Cash Transfers (GE), Siaya County

Target Population: All households across 653 rural villages in three subcounties taking part in an unconditional cash transfer program reported in [16].

Original Study Design:

The NGO GiveDirectly (GD) provides unconditional cash transfers to poor households; its cash transfer program was randomly assigned at the village level. In treatment villages, poor households meeting a basic means test (33 percent of households) were eligible for the program. Eligible households in treatment villages that were enrolled in the program received a large, unconditional cash transfer of about USD 1,000 (nominal) in a series of three payments over eight months via the mobile money platform M-Pesa. These transfers were rolled out across study villages, with households beginning to receive transfers in 2014-15. A total of 10,500 households received transfers. The NGO identified 653 villages across three subcounties in Siaya County for potential expansion for their unconditional cash transfer program in 2014, covering over 65,000 households and approximately 280,000 individuals. These were all rural villages in the three subcounties that had not previously been a part of GD's program. The study area was selected by the NGO due to its high poverty rates. In 2019, in preparation for a second endline survey, another household census was conducted and new households were incorporated into the sampling frame.

Sampling Frame: The sampling frame for Egger et al. (2019) was developed as follows: For our second endline, we augmented these 9,150 households with additional households identified in the 2019 census activity. We classify these new households into three groups: i) households that moved to the study area since our last census, ii) households that were newly formed from an existing household (most commonly children or siblings moving into their own household), iii) households that report having been in the study area at the time of our baseline census, but were not captured (this combines households that were actually present and missed with household misreporting). We include all households from group (ii) that split off of any of the 9,150 households in our original sample, as well as any split-offs from an additionally-drawn 18% of households not eligible for the cash transfer at baseline. In each village, we draw 24% of households belonging to groups (i) and (iii), but at least 1 household each. This provides a total sampling frame of 11,519 households.

COVID-19 Survey Design: Households targeted for inclusion in the second endline survey were included in two rounds of phone surveys, with the full sample targeted as part of each round.

Sampling Frame: Our total household sample for COVID-19 phone surveys

includes 11,519 households that were planned for surveys as part of the second in-person endline surveys.

Survey Dates: Round 1 of COVID phone surveys took place April 11–June 16, 2020. A second round was conducted June 18, 2020–September 2, 2020.

Sample size, tracking and attrition: 11,519 households.

Median survey time: 25 minutes.

Sampling Weights: Surveyed households are weighted by their 2019 census status in order to maintain population representativeness with our 2019 household census.

IRB Approval: UC Berkeley, Maseno University.

Kenya KEN3, Rural Households in Health Diaries project

Project Title: Enhancing Universal Health Coverage in Kenya through Digital Innovations: A Financial and Health Diaries evaluation study of I-PUSH

Target Population: Rural households with pregnant women or children under 4 years old in Kakamega (Khwisero subcounty) and Kisumu (Kisumu East and Seme subcounties).

Original Study Design: Initial baseline data was collected in-person in October/November with weekly diaries (in-Person) anticipated from December 2019 for a duration of 12 months. The study would conclude with an endline survey after those 12 months. In Kakamega, the study design entailed a randomized control trial to evaluate the impact of a mobile phone-based health insurance scheme. In Kisumu, the study was designed to evaluate free access to public care through a prospective longitudinal analysis.

Sampling Frame: The study population, drawn from low-income rural villages, consists of households with either a pregnant woman or a mother with children below four years old. First, 32 villages were randomly selected from the catchment areas of six health facilities, 24 in Kakamega and 8 in Kisumu. Next, in each village in Kakamega, ten households were randomly sampled from lists with households fulfilling the study eligibility criteria, resulting in 240 sampled households. In Kisumu, between 11 and 15 households per village were randomly selected resulting in 107 sampled households.

COVID-19 Survey Design: Phone surveys. After the onset of the first COVID-19

case in Kenya (mid-March 2020), the weekly diaries data collection transitioned from in-person interviewing to phone interviews.

Sampling Frame: Since weekly data collection was ongoing, the sampling frame for the phone surveys was the same as for the original study. In addition to the original survey modules, a special COVID-19 module was added in May 2020, including questions on symptoms of COVID-19, changes in health care utilization, and knowledge, attitudes and behaviours in response to COVID-19 preventive measures.

Survey Dates: October 18, 2019 to July 15, 2021

Sample size, tracking and attrition: Kakamega: 240 households, of which 44 dropped out between baseline and midline, while 57 were added to the sample, resulting in 253 households at the midline point. Kisumu: 107 households, of which 18 dropped out between baseline and midline, while 4 were added, resulting in a midline sample of 93 households.

Median survey time: N/A.

Sampling Weights: None.

IRB Approval: AMREF Ethics and Scientific Review Committee Kenya P679/2019 (August 8, 2019) with an extension granted on April 21, 2020.

Nepal

COVID-19 Experience

Case History:

- First COVID-19 case confirmed on January 23, 2020
- Second case confirmed on March 23, 2020
- Total cases: 662,570 as of July 15, 2021 [1]
- Total deaths: 9,463 as of July 15, 2021 [1]

Mobility Restrictions:

- January 28, 2020: Land border with China closed
- March 22, 2020: All international flights stopped
- March 23, 2020: Land border with India closed

- March 24, 2020: National lockdown takes effect. Movement outside the home banned except to purchase necessities or receive medical care. Motorized vehicles without prior permission banned from use. All transport services banned. Extended to July 21, 2020.
- August 11, 2020: Only passengers that have permission from the District Covid Crisis Management Centre and Local Administration will be allowed to travel from one district to another.
- September 21, 2020: Domestic passenger flights and long-distance public transport has resumed. International flights have been allowed from October 1, 2020.

Social Distancing:

- March 18, 2020: Gatherings of more than 25 people banned, including places of worship.
- August 11, 2020: Permission for daily prayers festivals observed among family members at home.

School Closures:

- March 19, 2020 - September 17, 2021: All classes and examinations suspended.

Social Protection Responses:

- The government provided food aid packages to an unspecified number of vulnerable households, with one source estimating that 70 - 95% of households in this category had received assistance [17].
- Plans for utility fee waivers and a public works program for individuals in the informal sector were announced, although there is limited data on how many people have benefitted from these schemes [2].

Economic Context

Seasonality and Food Security In 2019, Nepal produced record-level cereal crops, the latest in a series of four consecutive bumper harvests. The Nepal post-COVID survey was conducted several months before the start of the 2020 lean period.

However, the FAO remained concerned about food insecurity for approximately 15% of the Nepalese population [18].

Social Protection Nepal has over 80 social protection schemes in operation [19], with more than half of their spending going towards people who are elderly or disabled, and the rest distributed between a variety of employment and scholarship schemes [20]. While coverage is fairly high, at 28% of the population, the average program provides very low levels of benefits, at only $2 - $5 per month [20].

Nepal NPL, Agricultural Households in Western Terai

Project Title: Western Terai Panel Survey (WTPS)

Target Population: Rural households in the districts of Kailali and Kanchanpur.

Original Study Design: Initial baseline data was collected in-person in July of 2019, and 5 rounds of phone survey data were collected between August 12, 2019 and January 4, 2020.

Sampling Frame: The phone survey sample includes 2,636 rural households in the districts of Kailali and Kanchanpur, which represent the set of households that responded to phone surveys from an original sample of 2,935 households. This sample was constructed by randomly sampling 33 wards from 15 of the 20 sub-districts in Kanchanpur and selecting a random 97 villages from within those wards. At the time of baseline data collection in July of 2019, 7 of these 97 villages were dropped from the sample due to flooding. Households belong to the bottom half of the wealth distribution in these villages, as estimated by a participatory wealth ranking exercise with members of the village.

COVID-19 Survey Design: Phone surveys

Sampling Frame: Two phone surveys were fielded in April, 2020. The first included detailed questions on social distancing and more sparse data on other socioeconomic variables. This survey attempted to reach the universe of 1,820 households that had responded to at least one prior phone call in 48 villages, and successfully reached 1,419 households. The second survey included only sparse information on social distancing and more detailed questions on socioeconomic variables. This survey attempted to reach a random sample of 500 households across the remaining 42 villages, and successfully reached 408.

Survey Dates: April 1 to April 29, 2020

Sample size, tracking and attrition: 1,981 households

Median Survey Time: 27 minutes (COVID-19 survey) and 18 minutes (Socioeconomic survey)

Sampling Weights: None.

IRB Approval: Yale University IRB Protocol 2000025621

Nigeria

COVID-19 Experience

Case History:

- First confirmed case: February 27, 2020
- Total cases: 169,074 as of July 15, 2021 [1]
- Total deaths: 2,126 as of July 15, 2021 [1]

Mobility Restrictions:

- March 18, 2020: Travel ban on hot-spot countries.
- March 23-September 5, 2020: International flights banned.
- March 29, 2020 - May 18, 2020: Partial lockdowns in hot-spot states.
- April 20 - June 8, 2020: Domestic flights banned.
- April 27, 2020 - May 7, 2020: Nationwide ban on interstate movement.
- May 2, 2020: Nationwide overnight curfew. Revised to 12am-4am in September 3, 2020.
- June 2, 2020: Reopening of worship centers.
- July 1, 2020: Lifting of nationwide travel ban.

School Closures:

- March 26, 2020: Closure of all schools and higher education institutions.
- July 1, 2020: School resumption for graduating students.

- September 5, 2020: School resumption nationwide with COVID-19 guidelines in place.

Social Distancing:

- March 18, 2020: Gatherings of more than 50 people banned in hot-spot states.
- April 27, 2020: Mandatory use of face masks in public.

Social Protection Responses:

- March 23, 2020: Central Bank of Nigeria announced stimulus package for households, SMEs and health sector.
- March 27, 2020: Government reached out private donors to raise $330 billion.
- April 1, 2020: Federal Ministry of Humanitarian Affairs, Disaster Management and Social Development (FMHADMSD) announced free food rations to needy.
- April 1, 2020: Government announcement on cash transfer ($52) to poor registered in National Social Register.
- Arpil 6, 2020: Government reached out multilateral donors to raise $6.9 billion.
- May 7, 2020: Government raised $4.34 billion from domestic stock market to finance the budget.

Economic Context

Seasonality The NGA pre-COVID survey was conducted several months before the start of the 2020 lean period. However, the Boko Haram insurgency has led to heightened levels of displacement and food insecurity in the region. Nigeria alone accounts for 42% of the region's total number of acutely food-insecure people. In terms of absolute numbers, Nigeria ranked among the world's 10 worst food crises in 2019, with 5 million food insecure people [18].

Social Protection The Federal Government led social protection includes the following programmes:

- The National Social Safety Net Project (NASSP)

- In-Care of the Poor (COPE), which was funded initially through the MDGs-DRG fund, targeted at extremely poor households (those headed by a female, the elderly, physically challenged,fistula or HIV/AIDS patients) with children of school-going age;
- The health fee waiver for pregnant women and under-fives (funded by the MDGs-DRG and provided on a universal basis)
- The Community-Based Health Insurance Scheme (CBHIS) (re-launched in 2011)
- SURE - P MNCH, CSWYE programmes funded from the savings from the oil subsidy reform in 2013
- At the State level, social protection programmes cover a range of broad interventions, which are implemented by government Ministries, Departments and Agencies (MDAs) and/or funded by international donors.

However, only about 2% of Nigerians were enrolled in the country's major social security program, the National Social Safety Net Project (NASSP), and most other programs had significantly lower coverage. Throughout the COVID-19 crisis, coverage of social safety services remained low. Between March 2020 and March 2021, only 4% of households received federal, state, or municipal government assistance in the form of cash [21].

Nigeria NGA, Young Adults in Edo and Delta States, Nigeria

Project Title: Irregular Migration and Misinformation in Nigeria
Target Population: Young adults in Edo and Delta states, Nigeria
Original Study Design: Initial baseline data was collected in-person from March 9 to 23, 2020, followed by a Covid-19 phone survey carried out from May 20 to July 23, 2020. Follow-up data for this sample was collected by way of a phone survey from April 22 to May 21, 2021. Respondents that could not be reached by phone received an in-person follow-up visit between August 9 and September 1, 2021.

Sampling Frame: We georeferenced all residential structures in the states of Edo and Delta states from satellite imagery, drew enumeration areas, and randomly selected enumeration areas and structures for inclusion in our sample. Enumerators

then located structures using GPS-enabled devices, and randomly selected households within buildings. After obtaining the head of household's consent, enumerators completed a brief household survey module and roster using a tablet, and the device randomly selected an eligible individual from each household roster for an in-depth interview. Eligible individuals included all resident men and women aged 18 to 39.

COVID-19 Survey Design: Phone survey.

Sampling Frame: For the Covid-19 phone survey, we attempted to reach all individuals with whom a baseline interview had been conducted in March 2020.

Survey Dates: The Covid-19 phone survey took place from May 20 to July 23, 2020. Follow-up mental health data was collected by phone from April 22 to May 21, 2021, and in person from August 9 to September 1, 2021.

Sample size, tracking and attrition: Follow-up data is available for 628 individuals, out of a relevant baseline sample of 880 individuals.

Median Survey Time: The baseline survey included a 15-minute household module and roster and a 60-minute in-depth individual interview. The Covid-19 and follow-up surveys typically required 30 minutes.

Sampling Weights: None.

IRB Approval: IPA IRB Protocol 15057.

Rwanda

COVID-19 Experience

Case History:

- First confirmed case: March 14, 2020
- Total cases: 51,625 as of July 15, 2021 [1]
- Total deaths: 616 as of July 15, 2021 [1]

Mobility Restrictions:

- March 18, 2020: All commercial passenger flights halted.
- March 21, 2020: Travel between different cities and districts prohibited except for medical reasons or essential services. Borders closed except for goods and cargo and returning Rwandan citizens and legal residents.

- March 21, 2020 - May 4, 2020: Nationwide lockdown. Movement for essential services allowed.
- April 25, 2020: People in need of essential services must request clearance online and wait for approval before attempting movement.
- May 4, 2020: Curfew instituted between 8 pm to 5 am. Curfew changed to 9 pm to 5 am on May 18 and to 10 pm to 4 am on Oct 13, 2020 until Nov, 2021.
- June 2, 2020: Travel between provinces is allowed.
- July 8, 2020: Public and private businesses reopened with essential staff.

School Closures:

- March 16, 2020: Schools closed for four weeks. On April 30, 2020, fully opened in February 2021.

Social Distancing:

- March 8, 2020: Concerts and other public gatherings that bring many people together postponed.
- March 15, 2020: Places of worship closed.
- April 19, 2020: Face masks required in public and in multi-family compounds.
- April 30, 2020: Meetings in public and mass gatherings prohibited.
- June 16, 2020: Domestic tourism and international tourism for visitors traveling with charter flights resumed.
- June 30, 2020: Non-contact outdoor sports permitted.
- July 8, 2020: Religious weddings, burials and funeral gatherings are allowed with no more than 30 people.
- August 14, 2020: Public and private sector offices allowed to work in person with 50% capacity.

Social Protection Responses:

- March 28, 2020: Food distributions reaching 20,000 beneficiaries initiated in three districts of Kigali, starting with urban poor who cannot work and have no garden. Government fixed prices for 17 basic food items [18].
- May 4, 2020: People able to resume work will no longer receive food.
- Government added 56,000 new families to its existing Vision 2020 Umurenge Program (VUP) social protection scheme [2].

Economic Context

Seasonality The post-COVID survey was conducted during the lean season. The first 2020 harvest produced above-average yields. Flooding and landslides earlier in 2020 did not impact the first harvest and minimally impacted the anticipated second harvest. After exceptionally high bean and maize prices in December 2019, prices declined through February with the first harvest, increased during early pandemic closures, and then began to decrease again in May 2020 [18].

Social Protection Rwanda runs a variety of social protection programs, including public works and cash transfer programs, under the umbrella of its Vision 2020 Umurenge Program (VUP). Over a million people have benefitted from this program [22], approximately 7.5% of the population (Beegle, Coudousel & Monsalve 2018).

Rwanda RWA, Participants in 100WEEKS cash tranfer program

Project Title: 100WEEKS impact measurement.

Target Population: Women between 20-40 years of age living in poverty in Musanze, Rwanda.

Original Study Design: Baseline interview , 5 phone interviews, endline interview after 100 weeks.

Sampling Frame: Participants have been surveyed as part of the monitoring activities of a cash transfer program. The target group for the implementation of the cash transfer program were women between 20-45 years of age, who are in the lowest income quintile and are expected to have the ability to move out of poverty.

COVID-19 Survey Design: Phone surveys.

Sampling Frame: The sampling frame is the same as the original study design. Phone surveys were already a standard part of the monitoring efforts of 100WEEKS.

The face-to-face baseline interviews that are conducted at baseline were postponed during the 2-month lockdown in Rwanda, which meant the newly selected women had to wait to start the program.

Survey Dates: 25 June 2016 - 01 April 2021.

Sample size, tracking and attrition: 843 women were drop out.

Median Survey Time: For the face-to-face interviews the average survey time is 45 minutes, depending on the experience of the enumerator. The phone interviews take 10-25 minutes, depending on the survey round.

Sampling Weights: None.

IRB Approval: Vrije University Amsterdam - 20210413.1.wjs400 (June 7, 2021)

Sierra Leone

COVID-19 Experience

Case History:

- First confirmed case: March 31, 2020
- Total cases: 6,122 as of July 15, 2021 [1]
- Total deaths: 113 as of July 15, 2021 [1]

Mobility Restrictions:

- March 16, 2020: Imposed quarantine on all international travelers. Non-essential government and businesses directed to begin working from home.
- March 22, 2020: National and international flights halted.
- April 5, 2020 - April 11, 2020 : Lock down 1
- April 11, 2020: Start of dusk to dawn curfew (9pm-6am). Changed to 11pm-6am on June 24, 2020, and 11pm-5am on July 3, 2020. Inter-District travel banned.
- May 3-5, 2020: Lock down 2
- June 23, 2020: Inter-District travel restrictions lifted
- July 13, 2020: All worship gatherings were allowed.

Social Distancing:

- April 7, 2020: Limits on large gatherings, church attendance, closure of courts; Safaricom suspends mobile money transaction fees to encourage cashless transactions
- June 23, 2020: Mask wearing in public spaces mandatory

School Closures:

- March 31, 2020 - October 5, 2020: Closure of all schools and higher education institutions.

Social Protection Responses:

- April 19-21, 2020: 25 USD Cash transfers and 25 Kgs of rice to Persons with Disabilities during Lockdown 1 (April 2020)
- May 3-5, 2020: 25 kgs of rice to persons with disabilities during Lockdown 2 (May 2020)
- Emergency cash transfer for 29,000 petty traders starting in June 2020. They have also increased the value of the cash transfers provided to existing beneficiaries of the safety net program [2]. Estimates of the coverage of these cash transfers varies. [2] suggest that up to 14% of the population should benefit from these programs
- Lower-interest loans and tax deferments for SMEs, and cash transfers to vulnerable female heads of households.

Economic Context

Seasonality The post-COVID surveys were conducted during the lean season. The FAO had estimated that one million people would require food aid between March and May 2020, and in its May 2020 report assessed that 1.3 million could need food aid during the June-August 2020 period absent mitigation efforts [18].

Social Protection Sierra Leone launched its Social Safety Net program to support vulnerable households in 2013 [23]. Coverage of this program has been quite limited, reaching only 2.3% of the population [15]

Sierra Leone (SLE), Participants in the Sierra Leone Rural Electrification project

Project Title: Sierra Leone Rural Electrification (SLRE)

Target Population: Households in 195 rural towns across all 12 districts of Sierra Leone. Of these, 97 villages were selected to benefit from an electrification program.

Original Study Design:

Intervention: The Government of Sierra Leone (GoSL) in collaboration with United Nations Office for Project Services (UNOPS) and international donors is implementing the Rural Renewable Energy Project (RREP). In its first wave, during 2017, the project provided stand-alone solar photovoltaic powered mini-grids to 54 communities across the country. Construction of mini-grids in a further 43 towns is ongoing. In selected communities, engineers construct 6kW–36kW power mini-grids that provide reliable power year-round. Electricity is free for schools and clinics. Residential and commercial users can acquire connections from commercial operators.

Sampling Frame: Household data was collected in 195 towns across all 12 districts of Sierra Leone. The GoSL selected 97 towns with (planned) mini-grids. We use Propensity Score Matching to select 98 control communities. Within communities, respondents were randomly selected from a census roster stratified by occupation status of farmers, business owners and other occupations [47 percent, 47 percent and 7 percent]. In each village, the intended sample was 43 households (20 farmers, 20 business, 3 other). Data was collected during June–July (108 communities) and November–December 2019 (87 communities). If a household on our sampling list was not available on the village visit day, we had a randomly sampled list of replacement households to survey. The replacement household would be the same occupation as the sampled household would have been so the sample ratio of 20-20-3 still held in each community. Total baseline sample constitutes 3,230 respondents.

COVID-19 Survey Design: An additional round of survey data was collected during March - April 2021, in the same communities and under the same households. Total followup survey sample constitutes 2,808 respondents in 108 villages.

Sampling Frame: See above.

Survey Dates: First round data was collected during June–July (108 communities)

and November–December 2019 (87 communities). An additional round of survey data was collected during March - April 2021, in the same communities and under the same households.

Sample size, tracking and attrition: Total baseline sample constitutes 3,230 respondents, total followup survey sample constitutes 2,808 respondents. Attrition rate is 13%.

Median survey time: 33 minutes.

Sampling Weights: None.

IRB Approval: Sierra Leone Ethics and Scientific Review Committee (SLERC 2904202) and Wageningen University (24062020).

References

1. Mathieu E, Ritchie H, Rodés-Guirao L, Appel C, Giattino C, Hasell J, et al. Coronavirus Pandemic (COVID-19). Our World in Data. 2020.

2. Gentilini U, Almenfi M, Orton I, Dale P. Social Protection and Jobs Responses to COVID-19: A Real-Time Review of Country Measures. World Bank, Washington, DC. 2020. Available from: https://openknowledge.worldbank.org/handle/10986/33635.

3. Chowdhury A. Inclusive digital payments solutions for the garment sector workers in Bangladesh. Business Fights Poverty. 2020. Available from: https://businessfightspoverty.org/articles/inclusive-digital-payments-solutions-for-the-garment-sector-workers-in-bangladesh/.

4. The Business Standard. PM to launch disbursement of cash aid 2020. Available from: https://tbsnews.net/coronavirus-chronicle/covid-19-bangladesh/pm-launch-disbursement-cash-aid-50-lakh-families-tomorrow.

5. ILO. Social protection in Bangladesh. International Labour Organization, Geneva, Switzerland. 2020. Available from: https://www.ilo.org/dhaka/Areasofwork/social-protection/lang--en/index.htm.

6. Government of the UK. Congo travel advice. 2020. Available from: https://www.gov.uk/foreign-travel-advice.

7. IMF. Policy Responses to COVID-19. International Monetary Fund, Washington, DC. 2020. Available from: https://www.imf.org/en/Topics/imf-and-covid19/Policy-Responses-to-COVID-19#C.

8. Forku, R. Schools reopen in DR Congo amid COVID-19. Anadolu Agency, Ankara, Türkiye. 2020. Available from: https://www.aa.com.tr/en/africa/schools-reopen-in-dr-congo-amid-covid-19/1937210.

9. U.S. Embassy in the Republic of Congo. COVID-19 Information. 2020. Available from: https://cg.usembassy.gov/covid-19-information/.

10. FAO. GIEWS Country Brief. The Democratic Republic of the Congo. The Food and Agriculture Organization. 2020. Available from: https://reliefweb.int/sites/reliefweb.int/files/resources/COD_14.pdf.

11. Center for Preventive Action. Instability in the Democratic Republic of Congo. 2022. Available from: https://www.cfr.org/global-conflict-tracker/conflict/violence-democratic-republic-congo.

12. Oyunge P, Chebii M. Kenya enhances its cash transfer programmes in response to the COVID-19 pandemic. fsd Kenya. 2020. Available from: https://fsdkenya.org/blog/kenya-enhances-its-cash-transfer-programmes-in-response-to-the-covid-19-pandemic/.

13. Matiang'i F. Kazi Mtaani initiative has to succeed. Nation. 2020. Available from: https://nation.africa/kenya/blogs-opinion/opinion/kazi-mtaani-initiative-has-to-succeed-1910028.

14. National Social Protection Secretariat. National Safety Net Program. Available from: https://www.socialprotection.or.ke/social-protection-components/social-assistance/national-safety-net-program.

15. Beegle K, Coudousel A, Monsalve E. Realizing the Full Potential of Social Safety Nets in Africa. Agence Française de Développement and the World

Bank. 2018. Available from: http://documents1.worldbank.org/curated/en/657581531930611436/pdf/128594-PUB-PUBLIC.pdf.

16. Egger D, Haushofer J, Miguel E, Niehaus P, Walker M. General Equilibrium Effects of Cash Transfers: Experimental Evidence From Kenya. Econometrica. 2022. Available from: https://onlinelibrary.wiley.com/doi/full/10.3982/ECTA17945.

17. Franciscon I, Arruda P. COVID-19 and social protection in South Asia: Nepal. The International Policy Centre for Inclusive Growth. 2020. Available from: https://ipcig.org/pub/eng/OP452_COVID_19_and_social_protection_in_South_Asia_Nepal.pdf.

18. FAO. GIEWS Country Brief. The Food and Agriculture Organization. 2020. Available from: https://www.fao.org/giews/countrybrief/index.jsp.

19. Ghimire C. Social Protection in Nepal. Kathmandu Post. 2019. Available from: https://kathmandupost.com/columns/2019/08/08/social-protection-in-nepal.

20. Sijapati B. The Quest for Achieving Universal Social Protection in Nepal: Challenges and Opportunities. Indian Journal of Human Development. 2017. Available from: https://journals.sagepub.com/doi/abs/10.1177/0973703017696378?journalCode=jhda.

21. Lain J, Vishwanath T. The COVID-19 crisis isn't over for workers in Nigeria. World Bank Blogs. 2021. Available from: https://blogs.worldbank.org/africacan/covid-19-crisis-isnt-over-workers-nigeria.

22. World Bank. Reducing Vulnerability and Strengthening Inclusion in Rwanda Through Rural Development and Enhancing Social Protection. World Bank, Washington, DC. 2019. Available from: https://www.worldbank.org/en/results/2019/10/29/reducing-vulnerability-and-strengthening-inclusion-in-rwanda-through-rural-development-and-enhancing-social-protection.

23. FAO. Social Protection: Key to eradicate rural poverty and food insecurity in Sierra Leone. The Food and Agriculture Organization. 2019. Available from: http://www.fao.org/reduce-rural-poverty/news/detail-events/en/c/1258868/.
