# Supplementary material for: COVID-19 and mental health in 8 low- and middle-income countries: A prospective cohort study
Source: PLoS Med. 2023 Apr 6;20(4):e1004081. doi: 10.1371/journal.pmed.1004081 (PMC10079130; doi:10.1371/journal.pmed.1004081)
Supplement: S2 Appendix — (PDF) [file pmed.1004081.s008.pdf]

S2 Appendix. The STROBE Checklist

STROBE Statement—Checklist of items that should be included in reports of ***cohort studies***

	Item No	Recommendation	Page No
**Title and abstract**	1	(*a*) Indicate the study's design with a commonly used term in the title or the abstract<br>(*b*) Provide in the abstract an informative and balanced summary of what was done and what was found	(a)Page 1<br>(b)Pages 2 and 3
**Introduction**			
Background/rationale	2	Explain the scientific background and rationale for the investigation being reported	Introduction: 2. paragraph
Objectives	3	State specific objectives, including any prespecified hypotheses	Introduction: 3.paragraph
**Methods**			
Study design	4	Present key elements of study design early in the paper	Methods: 1. paragraph
Setting	5	Describe the setting, locations, and relevant dates, including periods of recruitment, exposure, follow-up, and data collection	Methods: 2. and 3. paragraphs
Participants	6	(*a*) Give the eligibility criteria, and the sources and methods of selection of participants. Describe methods of follow-up<br>(*b*) For matched studies, give matching criteria and number of exposed and unexposed	Methods: 2. paragraph
Variables	7	Clearly define all outcomes, exposures, predictors, potential confounders, and effect modifiers. Give diagnostic criteria, if applicable	Methods - Construction of main variables: 7., 8., 9.,10.,11. paragraphs
Data sources/ measurement	8*	For each variable of interest, give sources of data and details of methods of assessment (measurement). Describe comparability of assessment methods if there is more than one group	Methods: 10.paragraph and Data availability: 1. paragraph
Bias	9	Describe any efforts to address potential sources of bias	Methods- Estimation strategies per sample: 5. paragraph
Study size	10	Explain how the study size was arrived at	Abstract -Methods and Findings and Methods -Study design and participants: 1.paragraph
Quantitative variables	11	Explain how quantitative variables were handled in the analyses. If applicable, describe which groupings were chosen and why	Methods- Construction of main variables: 1. and 2. paragraphs
Statistical methods	12	(*a*) Describe all statistical methods, including those used to control for confounding<br>(*b*) Describe any methods used to examine subgroups and interactions<br>(*c*) Explain how missing data were addressed<br>(*d*) If applicable, explain how loss to follow-up was addressed<br>(*e*) Describe any sensitivity analyses	Methods- Empirical methodology:<br>(a) 1-4. paragraphs<br>(b) S5 section.<br>(c) Estimation strategies per sample section.
**Results**			

Participants	13*	(a) Report numbers of individuals at each stage of study—eg numbers potentially eligible, examined for eligibility, confirmed eligible, included in the study, completing follow-up, and analysed<br>(b) Give reasons for non-participation at each stage<br>(c) Consider use of a flow diagram	(a)Results section- figures and tables reports numbers of individuals
Descriptive data	14*	(a) Give characteristics of study participants (eg demographic, clinical, social) and information on exposures and potential confounders<br>(b) Indicate number of participants with missing data for each variable of interest<br>(c) Summarise follow-up time (eg, average and total amount)	(a)Methods- Study design and participants: 1.paragraph(Figure S2)<br>(b)Methods- Study design and participants: 3.paragraph
Outcome data	15*	Report numbers of outcome events or summary measures over time	(a)Methods- Study design and participants: 1.paragraph(Figure S2)
