# Supplementary material for: COVID-19 and mental health in 8 low- and middle-income countries: A prospective cohort study
Source: PLoS Med. 2023 Apr 6;20(4):e1004081. doi: 10.1371/journal.pmed.1004081 (PMC10079130; doi:10.1371/journal.pmed.1004081)
Supplement: S3 Appendix — (PDF) [file pmed.1004081.s009.pdf]

S3 Appendix. Analysis Plan

We did not post a public prospective analysis plan for this project. After exploratory analysis on the NPL sample but before receiving or analyzing other samples, we created an internal analysis plan. The plan included instructions for sample teams for supplying datasets, methods for selecting survey questions to include in our indices in samples where validated depression measures were not available. We also included our main specifications for the random-effects model to aggregate results across samples and our specification with individual fixed effects without additional controls used for BGD, DRC, NGA, SLE, as included in **S3 Fig**. In addition, based on our experience with the importance of seasonality in the NPL sample we planned on estimating equation (2) for samples with seasonal food security measures that pre-dated our mental health data.

We then received data from the majority of samples, generating a first version of **S2 Fig** and estimated the specification without controls (aggregated in **S3 Fig**) for 6 of our 10 samples. At this time we decided to update our analysis based on several facts: First, in the four of six samples with multiple pre-COVID survey waves we see clear evidence of secular pre-COVID trends in mental health. Second, aside from our NPL sample we had only been able to obtain supplementary seasonality data to extend our pre-period in the KEN2 sample and in no others, and therefore could not correct for seasonal trends using equation (2) in most samples. Third, we had been able to acquire over a full year of pre-COVID mental health data for three samples, which allowed better modeling options than we had originally considered.

In response to these observations in the data and the availability of longer pre-COVID panels, we estimated what we now believe to be our most credible model, equation (1), on the three samples that allowed for it. In the KEN3 sample, we see clear evidence of pre-COVID trends that appear to continue after the pandemic, but lacked the data necessary to adjust for it using equations (1) or (2). We decided to control lineary for these pre-trends and, because we only had 4 months of pre-pandemic data to estimate these trends, decided not attempt to estimate the longer-term effects of the pandemic by extrapolating this trend past 4 months post-Pandemic.

At this time we noticed that although our aggregate results were fairly robust, sample-specific estimates were model-dependent for some samples, particularly in the KEN3 sample where we lacked data to correct for pre-trends in our preferred methods. We also noticed that for the KEN3 sample we had a large density of survey observations near the onset of the pandemic that could be used in a more rigorous regression discontinuity design. We therefore then decided to stress-test our results by estimating an RD model in the samples where we had multiple pre and post-COVID survey waves within 3 months of the pandemic onset.

In response to reviewer comments, we (i) added heterogeneity in our estimates based on baseline characteristics of age, gender, and socioeconomic status and (ii) added a decomposition of the association between mental health and lockdowns and case-counts in our samples in to the **S5 Appendix**.
