# Supplementary material for: COVID-19 and mental health in 8 low- and middle-income countries: A prospective cohort study
Source: PLoS Med. 2023 Apr 6;20(4):e1004081. doi: 10.1371/journal.pmed.1004081 (PMC10079130; doi:10.1371/journal.pmed.1004081)
Supplement: S5 Appendix — (PDF) [file pmed.1004081.s011.pdf]

S5 Appendix. Relationship between Mental Health, COVID Cases and COVID Restrictions

In **S4 Fig**, we present estimates of the separate relationships between COVID-19 cases, the stringency of COVID-19 policy restrictions, and our unweighted depression index. These estimates were obtained as follows. First, in each of our six samples with multiple post-COVID survey rounds (COL, KEN1, KEN2, KEN3, NPL, RWA) we residualize our depression index, an index of the stringency of COVID-19-related policy restrictions in each country, and the inverse-hyperbolic sine of new COVID-19 cases in the country. We regress each of the three variables above on individual fixed-effects and the set of seasonality and time controls for each sample used in **Table 1**, and obtain the residuals from each regression. We then have $depression^r_{i,t,s}$, $stringency^r_{t,s}$, and $ihs(cases)^r_{t,s}$ which are residualized values of our depression index, policy stringency index, and inverse-hyperbolic sine of new COVID cases for individual i at time t in sample s. We then pool all of the data in our samples and estimate a random-effects model like the one below:

$$depression^r_{i,t,s} = \alpha_s stringency^r_{t,s} + \beta_s ihs(cases)^r_{t,s} + \epsilon_{i,t,s}$$

Where α_s and β_s are random-coefficients on policy stringency drawn from separate distributions:

$$\alpha_s \sim N(\mu_\alpha, \sigma^2_\alpha) \quad , \quad \beta_s \sim N(\mu_\beta, \sigma^2_\beta)$$

S4 Fig shows maximum likelihood estimates of μ_α and μ_β.
