# Supplementary material for: COVID-19 and mental health in 8 low- and middle-income countries: A prospective cohort study
Source: PLoS Med. 2023 Apr 6;20(4):e1004081. doi: 10.1371/journal.pmed.1004081 (PMC10079130; doi:10.1371/journal.pmed.1004081)
Supplement: S1 Table — (PDF) [file pmed.1004081.s012.pdf]

S1 Table. Description of household survey data samples used in the analysis

Country and COVID events	Projects	Households	Survey dates
Bangladesh<br>First case: March 8, 2020<br>Total cases (July 15, 2021): 1,071,774<br>Schools closed: March 17, 2020-September 13, 2021<br>Lockdown: March 26–May 30	BGD. Rohingya refugees from Myanmar: Refugee camp households in Bangladesh	3,517	Sept. 2019 - Oct. 2020
Colombia<br>First case: March 6, 2020<br>Total cases (July 15, 2021): 4,583,442<br>Schools closed: March 24, 2020<br>Lockdown: March 24, 2020–September 1, 2020	COL. Forcibly displaced primary caregivers in psychosocial support program: Caregivers of young children (ages 3-5) enrolled in public Early Childhood Development Centers in Tumaco, Colombia	662	March 2018 - Nov. 2020
Democratic Republic of the Congo<br>First case: March 6, 2020<br>Total cases (July 15, 2021): 45,211<br>Schools closed: March 28, 2020 - August 15, 2020<br>Lockdown: March 28, 2020 - August 15, 2020	DRC. Randomly selected Respondents from 80 villages in Nyangezi, Katana, and Walikale Health Zones in Eastern DRC	1,414	14 Sept. 2018 - 19 June 2021
Kenya<br>First case: March 13, 2020<br>Total cases (July 15, 2021): 191,020<br>Schools closed: March 20, 2020 - January 4, 2021<br>Curfew: March 27, 2020 - October 20, 2021	KEN 1. Participants in child health and human capital interventions: 6,500 Kenyans who were received one or more randomized health, skills training, and financial capital interventions during childhood and adolescence	2,677	12 Sep 2018 - 22 Feb 2020; 17 Apr 2020 - 2 Sep 2020
	KEN 2. Rural Households in NGO Cash Transfer Study: 653 rural villages taking part in an unconditional cash transfer program in Siaya County	7,585	30 May 2016 - 25 May 2017; 07 April 2020 - 02 Sept. 2020
	KEN 3. Rural households in Financial and Health Diaries study (i-PUSH): Households with pregnant women or children under 4 years old in Kakamega and Kisumu County	574	01 Oct. 2019 - 15 July 2021
Nepal<br>First case: January 23, 2020<br>Total cases (July 15, 2021): 662,570<br>Schools closed: March 19, 2020-September 17, 2021<br>Lockdown: March 24, 2020-July 21, 2020	NPL. Agricultural Households in Western Terai: Rural households in the districts of Kailali and Kanchanpur	1,372	27 Aug. 2019 - 22 Oct. 2020
Nigeria<br>First case: February 27, 2020<br>Total cases (July 15, 2021): 169,074<br>Schools closed: March 26, 2020 - September 5, 2021<br>Partial lockdowns: March 29, 2020 - May 18, 2020	NGA. Irregular Migration and Misinformation in Nigeria: Young adults in Edo and Delta states	217	09 March 2020 - 21 May 2021
Rwanda<br>First case: March 14, 2020<br>Total cases (July 15, 2021): 51,625 Schools closed: March 16, 2020 - February, 2021 Lockdown: March 21, 2020 - May 4, 2020	RWA. Participants in 100WEEKS cash tranfer program: Women in the lowest quantile of income in Musanze, Rwanda.	336	25 June 2016 - 01 April 2021
Sierra Leone<br>First case: March 31, 2020<br>Total cases (July 15, 2021): 6,122<br>Schools closed: March 31, 2020 - October 5, 2020<br>Lockdown: April 5–7, 2020, May 3–5, 2020	SLE. Participants in the Sierra Leone Rural Electrification project that installs solar mini-grids	2,808	01 June 2019 - 25 April 2021
