# Supplementary material for: COVID-19 and mental health in 8 low- and middle-income countries: A prospective cohort study
Source: PLoS Med. 2023 Apr 6;20(4):e1004081. doi: 10.1371/journal.pmed.1004081 (PMC10079130; doi:10.1371/journal.pmed.1004081)
Supplement: S2 Table — (PDF) [file pmed.1004081.s013.pdf]

S2 Table. Descriptive statistics

	Age			Gender (Female)			Std. depression index		
**Country**	N	Mean	SD	N	Percentage	SD	N	Mean	SD
Bangladesh	6.304	25,48	5.73	6.311	100,00%	0	6.311	6,70	6.57
Colombia	2.503	28,90	9.00	2.503	94,00%	0.23	2.503	-0,85	0.63
DRC	2.540	36,61	15.51	3.107	49,00%	0.50	3.133	-0,11	0.96
KEN1	5.405	33,08	2.49	5.405	55,00%	0.49	5.405	-0,43	1.19
KEN2	20.982	45,15	16.91	21.037	71,00%	0,45	24.970	0,05	0.62
KEN3	8.320	33,06	10.57	8.341	64,00%	0.47	8.342	0,69	0.95
Nepal	11.853	37,57	12.90	11.165	44,00%	0.49	13.143	-0,03	0.88
Nigeria	1.081	27,58	6.58	1.081	57,00%	0.49	1.081	-0,13	0.77
Rwanda	1.712	32,73	6.25	1.712	100,00%	0	2.568	-0,12	0.66
Sierra Leone	5.926	46,27	14.98	6.036	40,00%	0.49	6.036	-0,04	0.50
