# Supplementary material for: COVID-19 and mental health in 8 low- and middle-income countries: A prospective cohort study
Source: PLoS Med. 2023 Apr 6;20(4):e1004081. doi: 10.1371/journal.pmed.1004081 (PMC10079130; doi:10.1371/journal.pmed.1004081)
Supplement: S3 Table — This table presents question wording and answer options for mental health variables used in analysis. In BGD, COL, DRC, KEN3, and NGA samples, we followed the prescribed calculation method of each scale. In the remaining samples, we constructed indices of the available depression items as explained in the Data and Methods section. In BGD and KEN3, the total CES-D score (range: 0 to 80 and 0 to 30, respectively) is calculated by summing all the items. In COL, the SCL-90 score is calculated by dividing the raw score to number of questions in the depression domain (n = 13). In DRC, the final score is calculated by dividing the sum of the scores of all the items by 25 (the final score ranges from 1.00 to 4.00), where at least 22 items had to be answered for the assessment to be considered valid. In NGA, the total raw score, ranging from 0 to 25, is multiplied by 4 to give the final score, with 0 representing worst possible, whereas a score of 100 representing best possible quality of life. All scores are reverse coded and standardized by mean = 0, standard deviation = 1. The higher scores represents better mental well-being for each scale. (PDF) [file pmed.1004081.s014.pdf]

S3 Table. Question wording and answer options for depression measurement

Study	Question	Validated screening tool	Reference period
BGD	• How often did you feel so sad that nothing could cheer you up?<br>• I could not shake off the blues.<br>• I felt depressed.<br>• I felt sad<br>• I could not get going<br>• Nothing made me happy	CES-D-20, Cronbach's α (Scale reliability coefficient)= 0.948	Last week
COL	• Loss of sexual interest or pleasure<br>• Feeling low in energy or slowed down<br>• Thoughts of ending your life<br>• Crying easily<br>• Feeling of being trapped or caught<br>• Blaming yourself for things<br>• Feeling lonely<br>• Feeling blue<br>• Worrying too much about things<br>• Feeling no interest in things<br>• Feeling hopeless about the future<br>• Feeling everthing is an effort<br>• Feelings of worthlessness	SCL-90, Cronbach's α =0.855	Last 4 weeks
DRC	• Have you been bothered by these problems?<br>• Feeling low in energy or slowed down.<br>• Blaming yourself for things<br>• Crying easily<br>• Feeling sad or blue<br>• Feeling no interest in things/less interest in daily activities	HSCL-25, Cronbach's α = 0.943	Last 4 weeks
KEN1	• In past 7 days, how often have you felt depressed?	Subquestion from CESD-10	Last week
KEN2	• In the past week, I felt depressed and troubled in my mind.<br>• In the past week, I felt lonely.<br>• In the past 7 days, how often have you felt hopeful about the future?	Subquestions from CESD-10	Last week
KEN3	• Did you sleep well?<br>• Were you happy?<br>• Did you have trouble concentrating?<br>• Do you feel hopeful about the future?<br>• Did you feel that everything you did was an effort?<br>• Did you feel lonely?<br>• Did you feel depressed/stressed?<br>• Did you feel that you could not 'get going'?<br>• Were you bothered by things that don't usually bother you?<br>• Did you feel fearful?	CES-D-10, Cronbach's α = 0.773	Last week
NPL	• In the past 14 days how often have you felt sad or depressed?<br>• In the past 14 days, how often did you have difficulty sleeping at night?<br>• In the past 14 days how often have you felt stressed or worried?	N/A	Last 2 weeks
NGA	• I have felt cheerful and in good spirits<br>• I have felt calm and relaxed<br>• I have felt active and vigorous<br>• I woke up feeling fresh and rested<br>• My daily life has been filled with things that interest me	WHO-5 Well-being Index, Cronbach's α = 0.82	Last 2 weeks
RWA	In the past 7 days, were you/did you<br>• ...lost a lot of sleep because of worries?<br>• ...felt that you couldn't overcome your difficulties?<br>• ...able to face up to your problems?	N/A	Last week
SLE	• Feeling happy<br>• Feeling satisfied<br>• Facing difficulties/ feeling a lack of control over life	N/A	N/A

Notes. This table presents question wording and answer options for mental health variables used in analysis. In BGD, COL, DRC, KEN3 and NGA samples, we followed the prescribed calculation method of each scale. In the remaining samples we constructed indices of the available depression items as explained in the Data and Methods section. In BGD and KEN3, the total CES-D score (range: 0-80 and 0-30, respectively) is calculated by summing all the items. In COL, the SCL-90 score is calculated by dividing the raw score to number of questions in the depression domain (n=13). In DRC, the final score is calculated by dividing the sum of the scores of all the items by 25 (the final score ranges from 1.00 to 4.00), where at least 22 items had to be answered for the assessment to be considered valid. In NGA, the total raw score, ranging from 0 to 25, is multiplied by 4 to give the final score, with 0 representing worst possible, whereas a score of 100 representing best possible quality of life. All scores are reverse coded and standardized by mean=0, standard deviation=1. The higher scores represents better mental well-being for each scale.
