# Supplementary material for: COVID-19 and mental health in 8 low- and middle-income countries: A prospective cohort study
Source: PLoS Med. 2023 Apr 6;20(4):e1004081. doi: 10.1371/journal.pmed.1004081 (PMC10079130; doi:10.1371/journal.pmed.1004081)
Supplement: S4 Table — (PDF) [file pmed.1004081.s015.pdf]

S4 Table. Best Estimates from Each Sample (ICW Index)

	Season + Time Trend			Seasonal Food Security Ctrl		Time Control	Pre-Post Only			
	(1)	(2)	(3)	(4)	(5)	(6)	(7)	(8)	(9)	(10)
	RWA	COL	KEN1	KEN2	NPL	KEN3	BGD	NGA	SLE	DRC
0-2 months	-0.245	-0.0963	-0.705***	-0.304***	-0.196***	-0.0190				
	(0.378)	(0.0895)	(0.193)	(0.0156)	(0.0618)	(0.0478)				
2-4 months	-0.409		-0.873***	-0.271***	-0.0576	-0.152*				
	(0.430)		(0.183)	(0.0268)	(0.0626)	(0.0754)				
4-6 months	-0.113		-0.851***	-0.275***			0.0828**			
	(0.307)		(0.208)	(0.0400)			(0.0381)			
6-9 months	0.0421	-0.287***			0.0605**					
	(0.264)	(0.103)			(0.0292)					
9-12 months	-0.248									
	(0.323)									
12-15 months								-0.351***	-0.174***	0.263***
								(0.0829)	(0.0343)	(0.0482)
Year	-0.317**	0.127**	0.0172			0.878***				
	(0.150)	(0.0613)	(0.161)			(0.153)				
Seasonal Food				0.423***	0.136***					
Security				(0.105)	(0.0307)					
Observations	1532	2503	5405	24899	13143	8342	6311	1081	5978	3183

Standard errors in parentheses. * $p < .1$, ** $p < .05$, *** $p < .01$
