# Supplementary material for: COVID-19 and mental health in 8 low- and middle-income countries: A prospective cohort study
Source: PLoS Med. 2023 Apr 6;20(4):e1004081. doi: 10.1371/journal.pmed.1004081 (PMC10079130; doi:10.1371/journal.pmed.1004081)
Supplement: S5 Table — (PDF) [file pmed.1004081.s016.pdf]

S5 Table. Best Estimates from Each Sample (Factor Index)

	Season + Time Trend			Seasonal Food Security Ctrl		Time Control	Pre-Post Only			
	(1)	(2)	(3)	(4)	(5)	(6)	(7)	(8)	(9)	(10)
	RWA	COL	KEN1	KEN2	NPL	KEN3	BGD	NGA	SLE	DRC
0-2 months		-0.201**	-0.705***	-0.188***	-0.231***	-0.0527				
		(0.0907)	(0.193)	(0.0623)	(0.0652)	(0.0377)				
2-4 months	-0.342		-0.873***	0.0491	-0.0913	-0.187***				
	(0.630)		(0.183)	(0.108)	(0.0680)	(0.0601)				
4-6 months	-0.672		-0.851***	0.162			-0.0699*			
	(0.458)		(0.208)	(0.160)			(0.0392)			
6-9 months	-0.461	-0.336***			0.0443					
	(0.389)	(0.101)			(0.0307)					
9-12 months	-0.603									
	(0.480)									
12-15 months								-0.342***	-0.226***	0.177***
								(0.0835)	(0.0364)	(0.0537)
Year	0.541***	0.147**	0.0172			0.919***				
	(0.190)	(0.0599)	(0.161)			(0.104)				
Seasonal Food				1.811***	0.127***					
Security				(0.421)	(0.0331)					
Observations	1532	2503	5405	24838	13084	8342	6311	1076	5949	2891

Standard errors in parentheses. * $p < .1$, ** $p < .05$, *** $p < .01$
