# Supplementary material for: COVID-19 and mental health in 8 low- and middle-income countries: A prospective cohort study
Source: PLoS Med. 2023 Apr 6;20(4):e1004081. doi: 10.1371/journal.pmed.1004081 (PMC10079130; doi:10.1371/journal.pmed.1004081)
Supplement: S6 Table — (PDF) [file pmed.1004081.s017.pdf]

S6 Table. Pre-post differences in depression index (Factor weights)

	(1)	(2)	(3)	(4)	(5)	(6)	(7)	(8)	(9)	(10)
	BGD	COL	KEN 2	KEN 3	KEN 1	NPL	NGA	RWA	SLE	DRC
0-2 months		-0.0499	-0.0255	0.133***	-0.731***	-0.251***				
		(0.0572)	(0.0239)	(0.0434)	(0.0776)	(0.0726)				
2-4 months			0.183***	0.166***	-0.922***	-0.135*		0.832***		
			(0.0286)	(0.0471)	(0.0672)	(0.0754)		(0.204)		
4-6 months	-0.0699*		0.351***		-0.934***			0.334		
	(0.0392)		(0.0286)		(0.101)			(0.340)		
6-9 months		-0.0676				0.0484		0.256		
		(0.0452)				(0.0342)		(0.236)		
9-12 months								0.00953		
								(0.271)		
12-15 months							-0.342***		-0.226***	0.177***
							(0.0835)		(0.0364)	(0.0537)
Observations	6311	2503	24909	8342	5405	13084	1076	1532	5949	2891

Standard errors in parentheses

* $p < .1$, ** $p < .05$, *** $p < .01$
