# Supplementary material for: COVID-19 and mental health in 8 low- and middle-income countries: A prospective cohort study
Source: PLoS Med. 2023 Apr 6;20(4):e1004081. doi: 10.1371/journal.pmed.1004081 (PMC10079130; doi:10.1371/journal.pmed.1004081)
Supplement: S7 Table — (PDF) [file pmed.1004081.s018.pdf]

S7 Table. Pre-post differences in depression index (Inverse-covariance weights)

	(1)	(2)	(3)	(4)	(5)	(6)	(7)	(8)	(9)	(10)
	BGD	COL	KEN2	KEN3	KEN1	NPL	NGA	RWA	SLE	DRC
0-2 months		-0.00959	-0.455***	0.158***	-0.731***	-0.221***		-0.258		
		(0.0556)	(0.0227)	(0.0437)	(0.0776)	(0.0721)		(0.336)		
2-4 months			-0.309***	0.185***	-0.922***	-0.105		-0.310**		
			(0.0269)	(0.0468)	(0.0672)	(0.0727)		(0.140)		
4-6 months	0.0828**		-0.212***		-0.934***			-0.617***		
	(0.0381)		(0.0276)		(0.101)			(0.215)		
6-9 months		-0.0173				0.0697**		-0.370**		
		(0.0450)				(0.0341)		(0.178)		
9-12 months								-0.741***		
								(0.169)		
12-15 months							-0.351***		-0.174***	0.263***
							(0.0829)		(0.0343)	(0.0482)
Observations	6311	2503	24970	8342	5405	13143	1081	1712	5978	3183

Standard errors in parentheses

* $p < .1$, ** $p < .05$, *** $p < .01$
