# Supplementary material for: COVID-19 and mental health in 8 low- and middle-income countries: A prospective cohort study
Source: PLoS Med. 2023 Apr 6;20(4):e1004081. doi: 10.1371/journal.pmed.1004081 (PMC10079130; doi:10.1371/journal.pmed.1004081)
Supplement: S8 Table — (PDF) [file pmed.1004081.s019.pdf]

S8 Table. Pre-post differences in depression index (Unweighted index)

	(1)	(2)	(3)	(4)	(5)	(6)	(7)	(8)	(9)	(10)
	BGD	COL	KEN2	KEN3	KEN1	NPL	NGA	RWA	SLE	DRC
0-2 months		-0.0166	-0.353***	0.0489	-0.731***	-0.226***		-0.243		
		(0.0546)	(0.0226)	(0.0410)	(0.0776)	(0.0720)		(0.340)		
2-4 months			-0.179***	0.0467	-0.922***	-0.110		-0.283**		
			(0.0268)	(0.0442)	(0.0672)	(0.0729)		(0.142)		
4-6 months	0.0171		-0.0737***		-0.934***			-0.594***		
	(0.0389)		(0.0275)		(0.101)			(0.216)		
6-9 months		-0.0108				0.0669**		-0.351*		
		(0.0451)				(0.0341)		(0.180)		
9-12 months								-0.725***		
								(0.171)		
12-15 months							-0.356***		-0.198***	-0.241***
							(0.0831)		(0.0342)	(0.0477)
Observations	6311	2503	24970	8342	5405	13143	1081	1712	6036	3133

Standard errors in parentheses

* $p < .1$, ** $p < .05$, *** $p < .01$
