# Supplementary material for: COVID-19 and mental health in 8 low- and middle-income countries: A prospective cohort study
Source: PLoS Med. 2023 Apr 6;20(4):e1004081. doi: 10.1371/journal.pmed.1004081 (PMC10079130; doi:10.1371/journal.pmed.1004081)
Supplement: S9 Table — (PDF) [file pmed.1004081.s020.pdf]

S9 Table. Heterogeneity in Estimates by Gender

	(1)	(2)	(3)	(4)	(5)	(6)	(7)
	KEN1	KEN2	NPL	KEN3	DRC	NGA	SLE
0-4 months	-0.931***	-0.447***	-0.166*	-0.125			0
	(-3.50)	(-6.35)	(-2.22)	(-0.45)			
4+ months	-0.869**	-0.351*	0.0333		-0.254***	-0.416***	-0.237***
	(-2.74)	(-2.07)	(0.75)		(-3.66)	(-3.35)	(-5.35)
0-4 months × Female	0.225	0.144	-0.0472	-0.0467			
	(0.63)	(1.75)	(-0.40)	(-0.16)			
4+ months × Female	0.0855	0.117	0.0282		0.0273	0.105	0.0984
	(0.20)	(0.59)	(0.41)		(0.29)	(0.63)	(1.41)
Obs	5405	18503	11165	3906	3107	1081	6036
P: row 3 = 0 and row 4 = 0	0.749	0.0685	0.820	0.876	0.775	0.530	0.157

t statistics in parentheses

* $p < 0.05$, ** $p < 0.01$, *** $p < 0.001$
