# Supplementary material for: COVID-19 and mental health in 8 low- and middle-income countries: A prospective cohort study
Source: PLoS Med. 2023 Apr 6;20(4):e1004081. doi: 10.1371/journal.pmed.1004081 (PMC10079130; doi:10.1371/journal.pmed.1004081)
Supplement: S10 Table — (PDF) [file pmed.1004081.s021.pdf]

S10 Table. Heterogeneity in Estimates by Socioeconomic Status

	(1)	(2)	(3)	(4)	(5)	(6)	(7)	(8)	(9)	(10)
	COL	KEN1	RWA	KEN2	NPL	KEN3	BGD	DRC	NGA	SLE
0-4 months	-0.152	-0.790	0.330	-0.337***	-0.194**	-0.512*				
	(-1.15)	(-1.58)	(0.52)	(-6.43)	(-2.59)	(-2.08)				
4+ months	-0.253	-0.816	0.375	-0.281*	0.0347		-0.126*	-0.235**	-0.328*	-0.201***
	(-1.64)	(-1.42)	(0.58)	(-2.18)	(0.70)		(-2.25)	(-2.88)	(-2.30)	(-4.28)
0-4 months × High SES	0.00722	-0.192	-1.206	-0.00766	0.0708	0.562				
	(0.04)	(-0.33)	(-1.08)	(-0.10)	(0.64)	(1.79)				
4+ months × High SES	-0.119	-0.0981	-0.401	0.0337	0.0647		0.264***	-0.00761	-0.0482	0.00674
	(-0.60)	(-0.15)	(-0.55)	(0.19)	(0.95)		(3.41)	(-0.08)	(-0.27)	(0.10)
Obs	2503	3162	1617	18517	13123	3885	6311	3133	1075	6036
P: row 3 = 0 and row 4 = 0	0.783	0.923	0.559	0.887	0.571	0.0741	0.000660	0.940	0.784	0.921

t statistics in parentheses

* $p < 0.05$, ** $p < 0.01$, *** $p < 0.001$
