# Supplementary material for: COVID-19 and mental health in 8 low- and middle-income countries: A prospective cohort study
Source: PLoS Med. 2023 Apr 6;20(4):e1004081. doi: 10.1371/journal.pmed.1004081 (PMC10079130; doi:10.1371/journal.pmed.1004081)
Supplement: S11 Table — (PDF) [file pmed.1004081.s022.pdf]

S11 Table. Heterogeneity in Estimates by Age

	(1)	(2)	(3)	(4)	(5)	(6)	(7)	(8)	(9)	(10)
	COL	KEN1	RWA	KEN2	NPL	KEN3	BGD	DRC	NGA	SLE
0-4 months	-0.0912	-0.974***	-0.157	-0.431***	-0.183*	-0.156				
	(-0.66)	(-3.62)	(-0.31)	(-8.91)	(-2.39)	(-0.79)				
4+ months	-0.313*	-0.942**	-0.0379	-0.376**	0.0596		0.0584	-0.204**	-0.270*	-0.216***
	(-1.99)	(-2.95)	(-0.10)	(-3.18)	(1.22)		(0.99)	(-2.65)	(-2.31)	(-4.26)
0-4 months × High Age	-0.125	0.282	-0.208	0.205**	0.0378	-0.00486				
	(-0.68)	(0.79)	(-0.32)	(2.75)	(0.35)	(-0.02)				
4+ months × High Age	-0.00878	0.201	-0.142	0.288	-0.00686		-0.0764	-0.0687	-0.165	0.0309
	(-0.04)	(0.47)	(-0.28)	(1.58)	(-0.10)		(-0.98)	(-0.65)	(-1.00)	(0.45)
Obs	2503	5405	1712	18517	11853	3888	6304	2540	1081	5926
P: row 3 = 0 and row 4 = 0	0.754	0.720	0.939	0.0107	0.930	0.987	0.330	0.513	0.320	0.653

t statistics in parentheses

* $p < 0.05$, ** $p < 0.01$, *** $p < 0.001$
