# Supplementary material for: COVID-19 and mental health in 8 low- and middle-income countries: A prospective cohort study
Source: PLoS Med. 2023 Apr 6;20(4):e1004081. doi: 10.1371/journal.pmed.1004081 (PMC10079130; doi:10.1371/journal.pmed.1004081)
Supplement: Attachment — Submitted filename: Covid MH response letter.pdf [file pmed.1004081.s023.pdf]

Manuscript PMEDICINE-D-22-02441R1

Response to Editors and Reviewers

Dear Callam Davidson,

We appreciate the opportunity to submit a revised draft of the paper "The Effect of the COVID-19 Pandemic on Mental Health in Low and Middle Income Countries" for consideration for publication in the PLOS Medicine. We appreciate the time and effort you, editors and the reviewers took to provide thoughtful criticism and helpful revisions on our article. The editors' and reviewers' recommendations have been taken into account for the revision. Please see a detailed answer to each comment and corresponding changes below in blue. All page references are to the revised version of the document.

Best regards,
Mushfiq Mobarak

Editors' Comments to the Authors:

1. Substantial editing is required to align the manuscript with PLOS Medicine guidelines. In addition to the comments below, please review the instructions at https://nam12.safelinks.protection.outlook.com/?url=https%3A%2F%2Fjournals.plos.org%2Fplosmedicine%2Fs%2Fsubmission-guidelines&data=05%7C01%7Cahmed.mobarak%40yale.edu%7C2061405ea5d548d8bd6608daa145d2f4%7Cdd8cbebb21394df8b4114e3e87abeb5c%7C0%7C0%7C637999618538133417%7CUnknown%7CTWFpbGZsb3d8eyJWIjoiMC4wLjAwMDAiLCJQIjoiV2luMzIiLCJBTiI6Ik1haWwiLCJXVCI6Mn0%3D%7C3000%7C%7C%7C&sdata=jYmWYejKrJsVjDJ2gLcF%2BGJJgYg4ksgREBG5D8rIA9E%3D&reserved=0 and ensure your manuscript meets the requirements.

   Author response: Thank you for this note. We have edited the manuscript to reflect PLOS Medicine's guidelines and style requirements.

2. Please revise your title according to PLOS Medicine's style. Your title must be nondeclarative and not a question. It should begin with main concept if possible. "Effect of" should be used only if causality can be inferred, i.e., for an RCT. Please place the study design ("A randomized controlled trial," "A retrospective study," "A modelling study," etc.) in the subtitle (ie, after a colon).

Author response: Thank you for pointing this out. We have revised the manuscript title according to PLOS Medicine's style. We have replaced the title as **"COVID-19 and mental health: A prospective cohort study in eight Low- and Middle-Income Countries"**.

3. Please structure your abstract using the PLOS Medicine headings (Background, Methods and Findings, Conclusions).

Author response: In line with your request, we have restructured the abstract using the PLOS Medicine headings (Background, Methods and Findings, Conclusions).

4. Abstract Background: Provide the context of why the study is important. The final sentence should clearly state the study question.

Author response: In line with your recommendation, we have revised the abstract background as follows on page 3:

"The COVID-19 pandemic and associated mitigation policies created a global economic and health crisis of unprecedented depth and scale, raising the estimated prevalence of depression by more than a quarter in high-income countries. Low- and middle-income countries (LMICs) suffered the negative effects on living standards the most severely. However, the consequences of the pandemic for mental health in LMICs have received less attention. Therefore, this study assesses the association between the COVID-19 crisis and mental health in eight LMICs."

5. Abstract Methods and Findings:

* Please ensure that all numbers presented in the abstract are present and identical to numbers presented in the main manuscript text.
* Please include the study design, population and setting, number of participants, years during which the study took place, length of follow up, and main outcome measures.
* Please quantify the main results (with 95% CIs and p values).
* Please include the important dependent variables that are adjusted for in the analyses.
* In the last sentence of the Abstract Methods and Findings section, please describe the main limitation(s) of the study's methodology.

Author response: We have structured the Abstract Methods and Findings section following your comments on page 3.

6. Abstract Conclusions:

* Please address the study implications without overreaching what can be concluded from the data; the phrase "In this study, we observed ..." may be useful.
* Please interpret the study based on the results presented in the abstract, emphasizing what is new without overstating your conclusions.
* Please avoid vague statements such as "these results have major implications for policy/clinical care". Mention only specific implications substantiated by the results.

Author response: Thank you for the suggestions. We have updated the Abstract Conclusion section accordingly. Please see page 4.

7. The Data Availability Statement (DAS) requires revision. For each data source used in your study:

a) If the data are freely or publicly available, note this and state the location of the data: within the paper, in Supporting Information files, or in a public repository (include the DOI or accession number).

b) If the data are owned by a third party but freely available upon request, please note this and state the owner of the data set and contact information for data requests (web or email address). Note that a study author cannot be the contact person for the data.

Author response: Thank you for this comment. We have added a data availability statement in the methods section of the paper on lines 287-289:

"All replication files (de-identified data and code) are deposited in Harvard Dataverse. The replication file for the paper is available via this link https://doi.org/10.7910/DVN/RFODZC."

8. Please justify your response 'My study does not require an ethics statement' in the submission form. This does not appear to be correct.

Author response: We thank you for highlighting this response, which was indeed a mistake on our part. We have added an ethics statement in the methods section on lines 287-301, and we have corrected our response in the submission form accordingly.

9. We note that you conducted research or obtained samples in a foreign country. Did you consider including a local author as first or last author? If not, we recommend that

you consider doing so in line with ICMJE's authorship requirements (https://nam12.safelinks.protection.outlook.com/?url=https%3A%2F%2Fwww.icmje.org%2Frecommendations%2Fbrowse%2Froles-and-responsibilities%2Fdefining-the-role-of-authors-and-contributors.html&data=05%7C01%7Cahmed.mobarak%40yale.edu%7C2061405ea5d548d8bd6608daa145d2f4%7Cdd8cbebb21394df8b4114e3e87abeb5c%7C0%7C0%7C637999618538133417%7CUnknown%7CTWFpbGZsb3d8eyJWIjoiMC4wLjAwMDAiLCJQIjoiV2luMzIiLCJBTiI6Ik1haWwiLCJXVCI6Mn0%3D%7C3000%7C%7C%7C&sdata=hyCybRIfl0dNtiEBeoFCi2pUMInkAE%2BBgCwTQupWm1g%3D&reserved=0). PLOS has a parachute research policy which aims to promote collaboration and inclusivity in global health research. You are required to complete PLOS' questionnaire on inclusivity in global research and submit it with your revised paper. The policy and questionnaire can be found at https://nam12.safelinks.protection.outlook.com/?url=https%3A%2F%2Fjournals.plos.org%2Fplosone%2Fs%2Fbest-practices-in-research-reporting&data=05%7C01%7Cahmed.mobarak%40yale.edu%7C2061405ea5d548d8bd6608daa145d2f4%7Cdd8cbebb21394df8b4114e3e87abeb5c%7C0%7C0%7C637999618538133417%7CUnknown%7CTWFpbGZsb3d8eyJWIjoiMC4wLjAwMDAiLCJQIjoiV2luMzIiLCJBTiI6Ik1haWwiLCJXVCI6Mn0%3D%7C3000%7C%7C%7C&sdata=RIh9ffP5QHRGzPsN0O%2BPW4OzRTzlszA3OTQzVkeFhTk%3D&reserved=0.

Author response: Thank you for this recommendation, which we agree is an important consideration. We have followed the ICMJE's authorship requirements to guide our authorship decisions. We have included at least one author from each local team to make sure that our authors-list is representative and inclusive. In our research team, 11 out of 22 co-authors are citizens of a developing country, including the first co-author (Nursena Aksunger) and the last co-author (Ahmed Mushfiq Mobarak). We also added inclusivity in global health research form into the supplementary files and we mentioned the form in lines 303-304. We hope this information addresses your concerns about global collaboration and inclusivity.

10. Please include continuous line numbering throughout your manuscript.

    Author response: We have included the line numbering into the revised manuscript.

11. Citations should be in square brackets, and preceding punctuation. Please use the "Vancouver" style for reference formatting, and see our website for other reference guidelines https://nam12.safelinks.protection.outlook.com/?url=https%3A%2F%2Fjournals.plos.org%2Fplosmedicine%2Fs%2Fsubmission-guidelines%23loc-references&data=05%7C01%7Cahmed.mobarak%40yale.edu%7C2061405ea5d548d8bd6608daa145d2f4%7Cdd8cbebb21394df8b4114e3e87abeb5c%7C0%7C0%7C637999618538133417%7CUnknown%7CTWFpbGZsb3d8eyJWIjoiMC4wLjAwMDAiLCJQIjoiV2luMzIiLCJBTiI6Ik1haWwiLCJXVCI6Mn0%3D%7C3000%7C%7C%7C&sdata=nEIQut

%2FYVFpvHV%2B0ZRFkbMeh%2Bv3BjkH%2Fdu1JNrAqooA%3D&reserved=0

Author response: We have updated the reference formatting "APA style" to "Vancouver style".

12. Please remove the study results and/or conclusion from the Introduction.

    Author response: We have removed the study results and conclusion from the Introduction section, per your request.

13. Please conclude the Introduction with a clear description of the study question or hypothesis.

    Author response: Thank you for this note. We have added a description of the study question to the Introduction section. The revised text reads as follows on lines 44-54:

    "This study assesses the association between depression, the most prevalent mental disorder among adults, and the COVID-19 pandemic in LMICs. While a few studies have analyzed how mental health changed before and after COVID-19 in individual middle-income countries [...], our study is distinctive in that we track populations over multiple rounds, both pre-and post-pandemic, in eight countries from Sub-Saharan Africa (SSA), South-Asia, and South-America. Crucially, this allows us to explicitly adjust for time trends, and to take into account the independent effect of seasonal fluctuations in mental health related to lean and affluent periods driven by local crop and harvest cycles. In two countries, we collected high-frequency data on mental health that we can directly match to seasonal poverty and hunger, allowing us to investigate the importance of this adjustment in detail."

14. Your manuscript needs to be organised using the PLOS Medicine submission guidelines. Please see https://nam12.safelinks.protection.outlook.com/?url=https%3A%2F%2Fjournals.plos.org%2Fplosmedicine%2Fs%2Fsubmission-guidelines&data=05%7C01%7Cahmed.mobarak%40yale.edu%7C2061405ea5d548d8bd6608daa145d2f4%7Cdd8cbebb21394df8b4114e3e87abeb5c%7C0%7C0%7C637999618538133417%7CUnknown%7CTWFpbGZsb3d8eyJWIjoiMC4wLjAwMDAiLCJQIjoiV2luMzIiLCJBTiI6Ik1haWwiLCJXVCI6Mn0%3D%7C3000%7C%7C%7C&sdata=jYmWYejKrJsVjDJ2gLcF%2BGJJgYg4ksgREBG5D8rIA9E%3D&reserved=0 and organise your manuscript per the instructions found under 'Manuscript Organisation'.

    Author response: In line with your remark, we have organised the manuscript to reflect PLOS Medicine's submission guidelines.

15. Please label, cite, and present your Supporting Information as outlined here: https://nam12.safelinks.protection.outlook.com/?url=https%3A%2F%2Fjournals.plos.org%2Fplosmedicine%2Fs%2Fsupporting-information&data=05%7C01%7Cahmed.mobarak%40yale.edu%7C2061405ea5d548d8bd6608daa145d2f4%7Cdd8cbebb21394df8b4114e3e87abeb5c%7C0%7C0%7C637999618538133417%7CUnknown%7CTWFpbGZsb3d8eyJWIjoiMC4wLjAwMDAiLCJQIjoiV2luMzIiLCJBTiI6Ik1haWwiLCJXVCI6Mn0%3D%7C3000%7C%7C%7C&sdata=tC8muTErdzFsGINwwRDFV33Hzm4cUjZN7T3LC5TvfDQ%3D&reserved=0

    Author response: We have updated the Supporting Information section according to PLOS Medicine's guideline.

16. Much of what is presented in the Methods and, to some extent, the Results section (in terms of e.g., the relative merits and weaknesses of the different methodologies applied) would be better placed in the Discussion. Please use the Methods section to clearly describe the study conduct and analyses, in sufficient detail to allow other researchers to replicate your study. Please use the Results to describe your data without including interpretation/questions for the reader (this can be saved for the Discussion). Again, please review our submission guidelines for more information on PLOS Medicine style: https://nam12.safelinks.protection.outlook.com/?url=https%3A%2F%2Fjournals.plos.org%2Fplosmedicine%2Fs%2Fsubmission-guidelines&data=05%7C01%7Cahmed.mobarak%40yale.edu%7C2061405ea5d548d8bd6608daa145d2f4%7Cdd8cbebb21394df8b4114e3e87abeb5c%7C0%7C0%7C637999618538133417%7CUnknown%7CTWFpbGZsb3d8eyJWIjoiMC4wLjAwMDAiLCJQIjoiV2luMzIiLCJBTiI6Ik1haWwiLCJXVCI6Mn0%3D%7C3000%7C%7C%7C&sdata=jYmWYejKrJsVjDJ2gLcF%2BGJJgYg4ksgREBG5D8rIA9E%3D&reserved=0.

    Author response: We have re-organized the Methods, Results and Discussion sections as recommended, moving discussions of strengths and weaknesses of various methods as well the interpretation of the results to the Discussion section.

17. Your study is observational and therefore causality cannot be inferred. Please remove language that implies causality, such as 'Effects'. Refer to associations instead.

    Author response: Thank you for pointing this out. We concur with this observation. We have reframed the study results as associations and correlations throughout the manuscript.

18. Footnotes are not permitted. Please move the information into the main text or the reference list, depending on the content.

    Author response: As suggested by the Editor, we have moved the footnotes to the main text.

19. Please rename your 'Conclusion and Implications for Policy' section 'Discussion'.

    Author response: Thank you, we have done this on line 461.

20. Please present and organize the Discussion as follows: a short, clear summary of the article's findings; what the study adds to existing research and where and why the results may differ from previous research; strengths and limitations of the study; implications and next steps for research, clinical practice, and/or public policy; one-paragraph conclusion.

    Author response: Thank you for the comment. In line with the Editor's suggestion we have rewritten the Discussion section on lines 461-572, and structured it using the guidance provided above.

21. Please remove the List of Abbreviations.

    Author response: Thank you for the note. We have removed the List of Abbreviations from the manuscript.

22. Your Acknowledgements section contains details of the Ethical approval for the study - this information should be relocated to the Methods, alongside details of how informed consent was sought from participants (including whether this was written or verbal).

    Author response: Thank you for the comment. We have included the Ethical Approval details of each study to the Methods section on lines 290-299. Participants in each sample gave their verbal informed consent before each survey took place. In all but one sample - DRC - surveys were done by phone, and for the DRC sample, given the low literacy, consent was also verbal. We have added this information on lines 299-302.

23. Please remove the 'Authorship' section as this is captured as metadata based on the Submission Form responses.

    Author response: Thank you for the comment. We have removed the 'Authorship' section from the manuscript.

24. Please ensure that the study is reported according to the STROBE guideline, and include the completed STROBE checklist as Supporting Information. Please add the following statement, or similar, to the Methods: "This study is reported as per the Strengthening the Reporting of Observational Studies in Epidemiology (STROBE) guideline (S1 Checklist)."

The STROBE guideline can be found here: https://nam12.safelinks.protection.outlook.com/?url=http%3A%2F%2Fwww.equator-network.org%2Freporting-guidelines%2Fstrobe%2F&data=05%7C01%7Cahmed.mobarak%40yale.edu%7C2061405ea5d548d8bd6608daa145d2f4%7Cdd8cbebb21394df8b4114e3e87abeb5c%7C0%7C0%7C637999618538133417%7CUnknown%7CTWFpbGZsb3d8eyJWIjoiMC4wLjAwMDAiLCJQIjoiV2luMzIiLCJBTiI6Ik1haWwiLCJXVCI6Mn0%3D%7C3000%7C%7C%7C&sdata=IXvsDaOgDSe8E%2B2o5r7ZKAGoOrORiudUcUE%2BQlVoYJw%3D&reserved=0

25. When completing the checklist, please use section and paragraph numbers, rather than page numbers.

    Author response: Thank you for the note. We refer to the STROBE checklist in the revised manuscript on line 282 and we uploaded the STROBE checklist on the PLOS Medicine system. We made sure that we used section and paragraph numbers rather than page numbers.

26. Did your study have a prospective protocol or analysis plan? Please state this (either way) early in the Methods section.

a) If a prospective analysis plan (from your funding proposal, IRB or other ethics committee submission, study protocol, or other planning document written before analyzing the data) was used in designing the study, please include the relevant prospectively written document with your revised manuscript as a Supporting Information file to be published alongside your study, and cite it in the Methods section. A legend for this file should be included at the end of your manuscript.
b) If no such document exists, please make sure that the Methods section transparently describes when analyses were planned, and when/why any data-driven changes to analyses took place.

c) In either case, changes in the analysis-- including those made in response to peer review comments-- should be identified as such in the Methods section of the paper, with rationale.

For all observational studies, in the manuscript text, please indicate: (1) the specific hypotheses you intended to test, (2) the analytical methods by which you planned to test them, (3) the analyses you actually performed, and (4) when reported analyses differ from those that were planned, transparent explanations for differences that affect the reliability of the study's results. If a reported analysis was performed based on an interesting but unanticipated pattern in the data, please be clear that the analysis was data-driven.

Author response: Thank you for the comment. For this project we had an internal (non-published) analysis plan guiding the analysis of the data. We have added a short statement to the Methods section on lines 283-286, where we refer to a more elaborate description of our analysis plan and any modification thereof in the Supplementary Materials.

Reviewers' Comments to the Authors:

Reviewer #1

27. Excellent analysis and presentation. However, the document must expose the possible limitations of the study, for example, the diversity of instruments used to quantify depressive symptoms. These instruments have a substantial impact on the frequency of reported symptoms.

Author response: We thank the reviewer for the positive feedback, and for highlighting this limitation. We agree that this should be addressed in the discussion. We have added the following text to the Discussion section on lines 521-528:

"One potential limitation of our study is that the questions used to measure depression vary between surveys. However, previous research finds a very high correlation between different screening measures of depression [6]. Additionally, brief depression screening instruments are typically as accurate as long ones in LMICs [1], and previous research has validated the use of a single-item depression measure [50]. Our results are generally consistent across surveys despite these differences in how depression is measured, suggesting that the findings in this paper are not specific to one particular screening tool."

The Discussion section has also been expanded with two additional limitations, related to the relatively low number of pre- and post-COVID-19 survey waves in

samples (lines 529-534), and the need to include a larger number of population-based surveys from additional countries and continents to allow for an extrapolation of findings to LMICs in general (lines 538-545).

Reviewer #2

Thanks for the opportunity to review your manuscript. My role is as a statistical reviewer, so my review concentrates on the study design, data, and analysis that are presented. I have put general questions first, followed by queries relevant to a specific section of the manuscript (with a page/paragraph reference).

This study aims to quantify changes in levels of depression in people from LMICs associated with the COVID-19 pandemic. Data is aggregated from existing research in LMICs where measures of depression are collected. Levels of depression are taken from both validated and non-validated measures. Validated measures are standardised (to units of SD), un-validated ones have each relevant item standardised, then several different strategies to combine these items together (with and without weights). Each study has different periods of data collection - some studies allow for direct assessment of seasonal and underlying trend. In each study, a measure of the stringency of COVID-19 public health interventions is derived. A multilevel model (to incorporate repeated measurements of the same individuals over time) is used in each sub-sample to estimate difference between COVID and non-COVID periods (indicator variable), with effect of year and month (via binary indicators) also included. The estimate of difference between COVID and non-COVID periods is then included in a random-effects meta-analysis to provide an overall estimate of the impact of the COVID period on the depression score across all the sub-studies. Sensitivity analyses considering only sub-samples with a longer-times series, or restricted to the validated measurements of depression are considered. An interrupted time-series for sub-samples is also considered. The manuscript includes detailed and well-organised supplementary material, thank you, this makes reviewing much easier.

Author response: We thank you for the time taken to review the manuscript and provide constructive feedback. We have taken on board each of the points highlighted in your review, to which we respond in detail below, and where applicable we have also updated the manuscript in line with your recommendations. Thank you for your detailed review.

28. To my (non-expert) eyes, the questions in some of the depression measurements seem to be very different (Table A2). E.g. some of these concentrate on symptoms of depression (COL study), while in others it is effectively a self-report question rather than a depression scale (KEN1). My concern is whether some of these should be

included in the study if there is a so much difference between them (sensitivity analysis non-withstanding).

Author response: We thank the reviewer for raising this point, and we agree that this is something we should address more explicitly in the paper. We note that all measures are self-reports; what differs between samples is the screening tool used, and whether the questions jointly construct a validated depression score or instead are a subset thereof. We have added the following paragraph to the Discussion section on lines 521-528:

"One potential limitation of our study is that the questions used to measure depression vary between surveys. However, previous research finds a very high correlation between different screening measures of depression [6]. Additionally, brief depression screening instruments are typically as accurate as long ones in LMICs [1], and previous research has validated the use of a single-item depression measure [50]. Our results are generally consistent across surveys despite these differences in how depression is measured, suggesting that the findings in this paper are not specific to one particular screening tool."

29. The study and conclusions are described as generalizable to LMICs, while the data mostly comes from ad-hoc surveys in LMICs. Only 2/10 underlying samples have a population-based sampling frame, the rest are from specific samples within each country. The studies are based in countries from South Asia, Africa, and one South American country, and I think there are too many regions missed to be able to say that the overall study represents LMICs broadly.

Author response: We agree that this is a potential limitation of the study. We now explicitly mention that our study includes only eight countries in the title, the abstract and the introduction. Also, we have added the relatively small number of countries as a limitation of our study on lines 588-595, as follows:

"Finally, even though our study uses multiple datasets from three continents, its conclusion cannot necessarily be extrapolated to LMICs in general. Too many countries are missing in that respect, and eight out of our ten datasets are based on specific samples rather than on population-based sampling frames. Nevertheless, it is the first and only existing study covering a diverse set of LMIC regions with pre- and post-COVID-19 panel waves; and the robustness of the findings across individual settings is reassuring. Future research may uncover additional datasets as mental health becomes a more popular topic of inquiry."

30. In the main analyses and the ITS (discontinuity), what checks were made of the models, e.g. form of residuals, checks for influential observations, autocorrelation?

Author response: Thank you this question that we neglected to specify in the text. Our standard errors are clustered at the respondent-level and are robust to both heteroscedasticity (non-constant variance in residuals) and arbitrary correlation in outcomes within respondents, and we now clarify this in the text. We have also checked for sensitivity in our discontinuity results by varying the bandwidths (length of the time-series used) before and after the onset of the pandemic, without major changes in results. We have added this information on lines 197-199:

"We report heteroskedasticity-robust standard errors that also allow for arbitrary correlation between responses by the same participant using an Eicker-Huber-White-"sandwich" covariance estimator."

31. Much of the language used to describe the results e.g. 'the pandemic produced substantively and statistically significant increase in depression in LMICs' is causal. I would consider tempering the expression given that time-series are vulnerable to confounding, and detail some of the limitations of these methods in the discussion.

Author response: We agree with the reviewer's observation that we cannot infer causality from our data. Accordingly, throughout the manuscript, we have revised our language, and we now write up the methods and results as reflecting correlations and associations rather than causal effects.

32. P2, Paragraph 1. A change in units of standard deviation is difficult to interpret. Is there any context that could be added to the abstract (and elsewhere) to indicate what this translates in practice?

Author response: Thank you for this very good suggestion. To put our main SD results in perspective, we have now added a calculation showing that the main aggregate estimate of 0.29 SD is equivalent to moving from the 50th to the 63rd percentile in our median sample. This has been added to the Abstract and in the Discussion section on lines 471-472.

In addition, we have added an explicit comparison of our aggregate estimate with increases in depression symptoms in studies of high-income countries (HIC) (0.226 SD in a meta-analysis of 65 studies) and an explicit comparison of our aggregate estimates to the effects of asset transfer programs in LMIC settings (.14-.16 SD) ,

based on other studies that report results in standard deviation units. We have added this comparison in the Discussion section on lines 472-475 and 504-509.

33. P3, Paragraph 3. I would typically expect the introduction to introduce the context and research question rather than include results. Similarly, there is parts of the methodology in the introduction (P4).

Author response: Thank you for pointing this out. We have re-organized the Introduction according to the PLOS Medicine Guidelines in the revised version.

34. P7, Paragraph 1. How is the 'lean' season in each of the sub-studies defined?

Author response: Thank you for this relevant question. We have added the definition of the lean season for each country below Figure S1 in the Supporting Information which shows the calendar time for the lean season.

35. P8, Paragraph 8. To clarify, is each of the nine indicators a binary variable? i.e. there are 9 possible values for the index to take?

Author response: Thank you for the note. The Oxford Coronavirus Government Response Tracker (OxCGRT) project calculates a Stringency Index, a composite measure of nine of the categorical response metrics. The nine metrics used to calculate the Stringency Index are: school closures; workplace closures; cancellation of public events; restrictions on public gatherings; closures of public transport; stay-at-home requirements; public information campaigns; restrictions on internal movements; and international travel controls. The index on any given day is calculated as the mean score of the nine metrics, each taking a value between 0 and 100. We have added these details to our methods section on lines 139-145.

36. P9, Paragraph 5. What covariance structure was used for the repeated measurements of the same participants?

Author response: Thank you for requesting this. We have clarified that we allow for arbitrary correlation between responses by the same participant by using an Eicker-Huber-White-“sandwich” covariance estimator (Wooldridge, 2002) on lines 197-199.

37. Were survey weights from the population-based studies used in these analyses?

Author response: We do not have the survey weights for the population-based studies in our dataset, so these were not used in the analysis.

38. P10, Paragraph 2. Will this approach detect a secular trend in depression occurring within 1 year, or will that all be absorbed into the fixed effects for month across all the years of data?

Author response: We appreciate this question for clarification of our methods. These trends will be absorbed into fixed effects for months. As a result, our method will adjust for secular trends in depression occurring within one year when estimating changes in depression associated with COVID-19. In simple terms, we compare depression levels in a given month post-COVID-19 with depression levels in that same month pre-COVID-19 plus any year-on-year trend that we estimate from our year-on-year data (if available). For instance, in COL, October 2018 and October 2019 mental health levels are higher than average mental health levels in 2018 and 2019, respectively. In addition, mental health levels in March and October of 2019 are higher than mental health levels in March and October 2018, respectively. Therefore, we compare observed mental health levels in October 2020 to mental health levels in October pre-COVID-19 plus the average year-on-year increases from 2018 to 2019 in our COL sample. The difference is the estimate we obtain for the association between mental health and COVID-19. We have briefly clarified this in the manuscript on lines 170-173 as follows:

"So, for each sample separately, we compare depression levels in a given month post-COVID-19 with depression levels in that same month pre-COVID-19 (if available) plus any year-on-year trend that we estimate from our year-on-year data (if available). The sample-specific strategies are described in detail below."

39. P11, Paragraph 4. What software was used for the analyses?

Author response: Thanks for pointing this out. In our analysis 'Stata 15' is used as a software. We have included this information in the revised manuscript in line 279.

40. P17, Paragraph 1. Much of this material should be moved to a method section.

Was a sufficient number of time-points (e.g. 8 is often suggested) of data available before and after lockdown in each study?

Author response: We have moved the description of the methods to the Methods section, as suggested.

The number of time-points or survey waves ranges between 2 (one before and one after) and 17, with an average of 7.1 across samples. We have added this information to the abstract and in the Methods section on lines 76-77.

We acknowledge that a larger number of time points would have strengthened our analysis. This is a limitation to our analysis that we now briefly mention in the abstract, and more extensively in the Discussion section on lines 529-538, as follows:

"Another limitation is the small number of pre- and post-COVID-19 surveys available in most of our samples. Ideally, we would have had multiple rounds of pre-pandemic data for all samples to more fully account for seasonal effects and other time trends. Likewise, additional post-pandemic survey waves covering a longer time-period would have allowed us to examine the post-COVID-19 dynamics and recovery processes in greater depth. This underscores the importance of collecting long-running panels in LMICs. Such data can greatly enhance countries' epidemic preparedness, providing governments with crucial and timely information during health crises on the consequences of mitigation policies and the need for targeted support."

41. P18, Table 2. The trend line in these analyses extends beyond available data - e.g. with only a short period of data from KEN1 the post-COVID prediction line extends out 10 times the extend of available post-change data. These should be revised to the prediction line ends when there is no more data.

    In the post-onset time-period, there is also a line fitted from only two points in time for the Colombia_NW sub-sample. I understand that these points are averages of the sample at each time, but there are serious risks to trying to infer a slope change from only two time-points even if there are many measurements at each time.

    Author response: We thank you for pointing out that the trend line was extending too much beyond the available data. We have adjusted the figure such that the prediction line ends at the end of the data.

    We thank you for noting that, given the discrete nature of survey waves in each sample, the effective sample size for these trends is small and we have bunching in our running variable. This is a limitation of this analysis, and we now highlight this in our discussion section on lines 529-534.

Reviewer #3

This paper presents data from ten longitudinal pre-/post COVID-19 data sets spanning eight countries to examine changes in mental health from upward of 21,000 people in Low and Middle Income Countries (LMICs). Using creative and careful modelling, the authors combine impressive data from understudied regions to demonstrate the significantly negative change in mental health in the first few (~4) months of the pandemic, which reduces somewhat overtime. This trend is most noticeable when accounting for the strong seasonal variation in mental health that is shaped by food production patterns.
I thought there was much to be appreciated in this work. First, this paper provides an extremely important contribution to the literature by shedding light on the mental health changes in LIMCs during COVID-19 onset. As the authors note, we have learned much about mental health fluctuations during the pandemic in wealthy nations but the data are sparse elsewhere. Thus this fills a dire hole in our understanding of mental health under the COVID-19 pandemic. Second, I appreciated the thoughtful aggregation of the data and careful analytic strategy that modelled each country with the best information available, but also considered a generic approach for consistency. Third, as the authors make clear, attention paid to the local context and season patterns is critical to understand mental health changes.

Results seem clear and consistent with most of the findings I have read to date, and meta-analyses in the literature. Depression levels peaked soon after COVID-19 onset and began to return (although not fully cover) to pre-pandemic levels in most countries several months later.

> **Author response**: We thank you for the time taken to review the manuscript and provide constructive feedback. We have taken on board each of the points highlighted in your review, to which we respond in detail below, and where applicable we have also updated the manuscript in line with your recommendations. Thank you for your detailed review.

My questions primarily have to do with whether greater precision can be inferred from this impressive database:

42. 1. The authors speak to mental health changes from pre- to post-COVID-19 but is it possible to distinguish this further? For instance, are respondents reporting higher levels of depression in response to higher local case counts, higher deaths, greater policy stringency, or disruption from daily life? The authors briefly mention the importance of these alternative factors but I don't see them included in the models.

Author response: Thank you for these very good suggestions. We have included an estimation with respect to the "COVID-19 case counts" and the "COVID-19 stringency index" comparison per country in Figure S5. In the revised version we have provided a detailed explanation for how COVID-19 case counts and strictness of policy measures correlated with changes in mental health under the subsection "Heterogeneity and Decomposition of Results". We find that these two potential drivers of mental health problems equally contribute to our overall results; the coefficient estimate of a 1 SD change in policy stringency is roughly 3 times the magnitude of the estimate on a 1 SD change in (log) new COVID-19 cases. We have added these findings to the Results section on lines 426-441.

43. 2. The authors demonstrate the importance of appropriately capturing time/season variations in agricultural food cycles that impact food security at-scale. I wondered: Do these findings parallel at the individual level, such that people with lower incomes/greater food or work precarity report larger variations in mental health responses? More broadly, what heterogeneity is seen in the data? Are parents, elderly, the young, females/males at greater risk for depression, perhaps due to greater vulnerability?

Author response: This too is a very useful suggestion. We have incorporated your recommendation into our analysis. We show the results of the heterogeneity analyses in Tables S8-S10, and describe them in the Results section on lines 427-430. We do not find systematic evidence of heterogeneity by age, gender or socio-economic status.

44. 3. I notice in the appendix that some countries provided various social protection efforts, such as cash transfers to various industries. Could this provide a within-country robustness check, wherein recipients of such transfers or bursaries report better mental health than would be expected otherwise?

Author response: Thank you for this interesting suggestion. It would have been very useful to explore this aspect. However, this is not possible in our study due to data limitations; we do not have information about whether respondents were recipients of any social support program or not.

45. 4. Are the authors able and willing to benchmark changes in mental health observed in the LIMC to those observed elsewhere in higher income countries? It could be helpful to know if the observed increase in depression was larger in size in LIMCs to potentially garner greater aid.

Author response: This too, we agree, is a very useful idea. As suggested by the reviewer, we have compared the main results of this study to previous studies which assess changes in mental health observed in the high-income countries after the onset of the COVID-19 pandemic.

We have added a discussion of this comparison on lines 504-514 as follows:

"Our primary finding is consistent with the results from HIC studies, which mostly find a significantly negative association between COVID-19 and mental health, including a meta-analysis of sixty-five longitudinal cohort studies that compare mental health before versus during the COVID-19 pandemic (standardized mean change in depression 0.226 [95% CI: .109 to .343]) [38]. It is also in line with findings from a pre-post COVID-19 analysis which used helpline calls in HICs as a proxy for mental well-being [10]. Other individual country studies from Germany [7], United Kingdom (0.19 SD [95\% CI: 0.17, 0.21]) [34], [33, 16], United States (0.27 SD (95\% CI [0.23,0.31], $p < .001$)) [15] and Canada [36] also demonstrate worsened mental health problems during the COVID-19 pandemic. The effect size we estimate for LMICs in our meta-analysis [95\% CI .109 to .343] is very similar to the estimates reported for HICs.

46. 5. Along similar lines, I found the recommendations offered at the very end of the paper to be brief and broad. While training a fleet of paraprofessionals may be one strategy, I wondered whether any other more targeted suggestions could be provided based uniquely on the current data re: scope and timing of threat, and/or heterogeneity in response.

Author response: In the revised "Discussion" section, we now include broader discussions about the needs in LMICs, and how that context varies from high-income countries. We also discuss implications of our specific findings regarding seasonality in mental health that fluctuates with crop cycles in agricultural areas.
